# Pro-cognitive restoration of experience-dependent parvalbumin inhibitory neuron plasticity in neurodevelopmental disorders

**DOI:** 10.21203/rs.3.rs-5624085/v1

**Published:** 2025-01-16

**Authors:** Yu-Tzu Shih, Jason Bondoc Alipio, Zin-Juan Klaft, Nathaniel Green, Lai Ping Wong, Ruslan Sadreyev, Jung Ho Hyun, Chris Dulla, Amar Sahay

**Affiliations:** 1 Center for Regenerative Medicine, Massachusetts General Hospital, Boston, Massachusetts, USA; 2 Harvard Stem Cell Institute, Cambridge, Massachusetts, USA; 3 Department of Psychiatry, Massachusetts General Hospital, Harvard Medical School, Boston, Massachusetts, USA; 4 BROAD Institute of MIT and Harvard, Cambridge, Massachusetts, USA; 5 Department of Neuroscience, Tufts University School of Medicine, Boston, MA, USA; 6 Department of Molecular Biology. Massachusetts General Hospital, Harvard Medical School, Boston, Massachusetts, USA; 7 Department of Brain Sciences, Daegu Gyeongbuk Institute of Science and Technology, Daegu, South Korea

## Abstract

The hippocampus forms memories of our experiences by registering processed sensory information in coactive populations of excitatory principal cells or ensembles^1–3^. Fast-spiking parvalbumin-expressing inhibitory neurons (PV INs) in the dentate gyrus (DG)-CA3/CA2 circuit contribute to memory encoding by exerting precise temporal control of excitatory principal cell activity through mossy fiber-dependent feed-forward inhibition^4–13^. PV INs respond to input-specific information by coordinating changes in their intrinsic excitability, input-output synaptic-connectivity, synaptic-physiology and synaptic-plasticity^9,13–17^, referred to here as experience-dependent PV IN plasticity, to influence hippocampal functions. PV IN impairments in early life, when neural circuitry is highly sensitive to experience-dependent refinement, are thought to result in imbalanced excitation and inhibition, impaired cognition, network hyperexcitability and seizures: hallmarks of neurodevelopmental disorders (NDDs) such as Autism Spectrum Disorder and epilepsy^18–20^. Discovery of transcriptional regulators of experience-dependent PV IN plasticity in the adult hippocampus may permit reversal of these developmental impairments. Here, in a screen designed to capture the PV IN intrinsic program induced by increased mossy fiber inputs, a trigger for experience-dependent PV IN plasticity, we identify the homeobox gene *Meis2*^21^ as a regulator of experience-dependent PV IN plasticity gene (XPG) in the adult DG-CA3/CA2 circuit. We found that a significant number of upregulated XPGs also exhibit haploinsufficiency in ASDs, epilepsies, and schizophrenia. We demonstrate that virally-mediated rescue of experience-dependent *Meis2* upregulation in CA3/CA2 PV INs in a NDD risk mouse model in adulthood is sufficient to restore experience-dependent PV IN plasticity, spatial and social memory, ensemble specificity, suppression of network hyperexcitability and seizures. Together, these findings suggest that experience-dependent PV IN plasticity is a convergent mechanism for NDD risk genes that can be re-instated in adulthood to reverse developmental deficits in circuitry, network excitability and cognition.

The hippocampus plays a crucial role in formation, storage and retrieval of memories, allowing for calibration of motivated and defensive behaviors^2^. During the early postnatal period, experience refines hippocampal circuitry to influence cognitive trajectory^22^, disruption of which results in maladaptive neural circuit functions such as cognitive impairments and epilepsy that characterize different NDDs, including ASDs. The identification of ultra-high confidence genetic risk factors for NDDs underscores the need to understand how experience and genetic risk conspire to compromise experience-dependent mechanisms instrumental for critical hippocampal functions.

Fast-spiking parvalbumin-expressing inhibitory neurons (PV INs) in the hippocampal dentate gyrus (DG)-CA3/CA2 circuit influence encoding, storage, retrieval and routing of memories through activity-dependent regulation of CA3/CA2 principal cell perisomatic inhibition^4–12^. PV IN mediated feed-forward inhibition of CA3/CA2 principal cells dictates their spiking, synchronization of principal cell activity to form ensembles and the generation of network oscillations to mediate intra-hippocampal and inter-regional communication in hippocampal-cortical-subcortical networks^3,11,23,24^. To exert these effects on circuitry and network properties, PV INs cell-autonomously coordinate experience-dependent changes in their intrinsic properties. These include structural reorganization of axonal arborizations and perisomatic synapses in CA3/CA2, regulation of feed-forward inhibition of CA3/CA2, and synaptic plasticity^9,10,13–17^: collectively referred to here as experience-dependent PV IN plasticity. Loss of PV IN functions in NDDs including ASDs, schizophrenia and epilepsies may arise from genetic risk factors that impair experience-dependent refinement of PV IN mediated inhibition during the early postnatal period^18–20,25,26^.

Transcription factors and epigenetic regulators co-ordinate changes in gene expression underlying synaptic physiology, synaptic and structural plasticity and input-output connectivity to mediate experience-dependent inhibitory and excitatory neuron plasticity. We know a substantial amount about molecular mechanisms that regulate cortical PV IN identity and experience-dependent plasticity^14,19,27–29^ and less about developmental regulators of hippocampal PV IN properties^15,30,31^. In sharp contrast, evidence for transcription factors and epigenetic regulators that control experience-dependent PV IN plasticity in the adult hippocampus is scarce.

PV INs in CA3/CA2 retain experience-dependent plasticity in adulthood. Specifically, learning^6,10^ and social experience^9^ increase mossy fiber excitatory synaptic inputs onto PV INs to trigger experience-dependent PV IN plasticity–subsequently leading to increased feed-forward inhibition in DG-CA3 and DG-CA2^9^. Increased mossy fiber excitatory drive onto PV INs increases intrinsic excitability, inhibitory synapses onto CA3 and CA2, and perisomatic inhibition of CA3 and CA2 principal cells to support spatial and social memory^9,10^. At a network level, DG recruitment of PV IN-mediated inhibition of CA3/CA2 principal cells promotes stable and context-specific neuronal ensembles in hippocampal-cortical networks and network oscillations during memory consolidation ^10,11^. Building on these observations, we designed and implemented a screen to identify regulators of experience-dependent PV IN plasticity genes (XPGs) in the adult CA3/CA2 circuit. We discovered a suite of XPGs, including transcription factors and epigenetic regulators, that are encoded by ultra-high confidence risk genes for NDDs and epilepsies. We show that the homeobox transcription factor *Meis2*^21^ is a regulator of experience-dependent PV IN plasticity. Further, we used PV IN enhancer-driven AAV-Meis2 viral vectors to test the hypothesis that restoration of experience-dependent PV IN plasticity in the adult hippocampus in a prototypical NDD risk model is sufficient to reverse developmental deficits in circuitry, ensemble specificity, network excitability, seizures and cognition.

## Screen for experience-dependent PV IN plasticity genes in adult CA2/CA3

**Extended Data Fig. 1.**
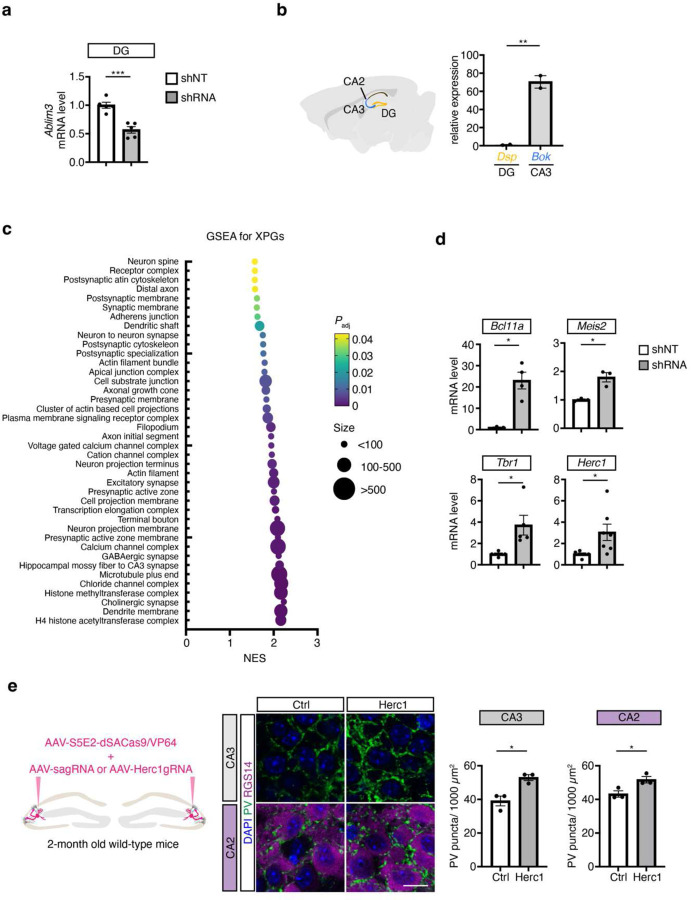
Supporting data for screen for regulators of experience-dependent PV IN plasticity. **a**, Quality control data: Validation of *Ablim3* downregulation in DG of *PV*^*Cre*^:*Rpl22HA*^*f/f*^ mice injected with lentiviruses expressing *Ablim3* shRNA-GFP (vs. non-targeting shRNA, shNT-GFP) into DG (N = 5 mice per group) by qRT-PCR. **b**, Enrichment of CA3/CA2 tissue: qRT-PCR for *Dsp* (DG enriched) and *Bok* (CA3 enriched) expression in CA2/CA3 tissue from *PV*^*Cre*^:*Rpl22HA*^*f/f*^ mice (N = 2 mice per group). **c**, Gene Set Enrichment Analysis (GSEA) of ribosome-associated RNAs isolated from CA3/CA2 PV INs (FDR < 0.05). **d**, qRT-PCR data for *Bcl11a* (N = 3 mice for shNT, 4 for shRNA group), *Meis2* (N = 3 mice per group), *Tbr1* (N = 5 mice per group) and *Herc1* (N = 6 mice for shNT, 7 for shRNA group) expression levels in CA2/CA3 PV INs of *PV*^*Cre*^:*Rpl22HA*^*f/f*^ mice injected with lentiviruses expressing *Ablim3* shRNA-GFP or non-targeting shRNA (shNT-GFP) into DG. **e**, Left: Experimental design. AAV expressing S5E2 enhancer with dSaCas9 combined with either scrambled gRNA (sagRNA) or Herc1 gRNA were injected into the CA2/CA3 of 2-month-old wildtype mice 2 weeks prior to perfusion. Right: representative images and quantification of PV^+^ puncta density in CA2 (RGS14^+^ labeling) and CA3 (N = 3 mice for each group). Scale bar, 10 μm. *p < 0.05; **p < 0.01; ***p < 0.001 using two-tailed unpaired *t* test with Welch’s correction. All experiments were performed in male and female mice, and all data are displayed as mean ± SEM.

**Fig. 1.**
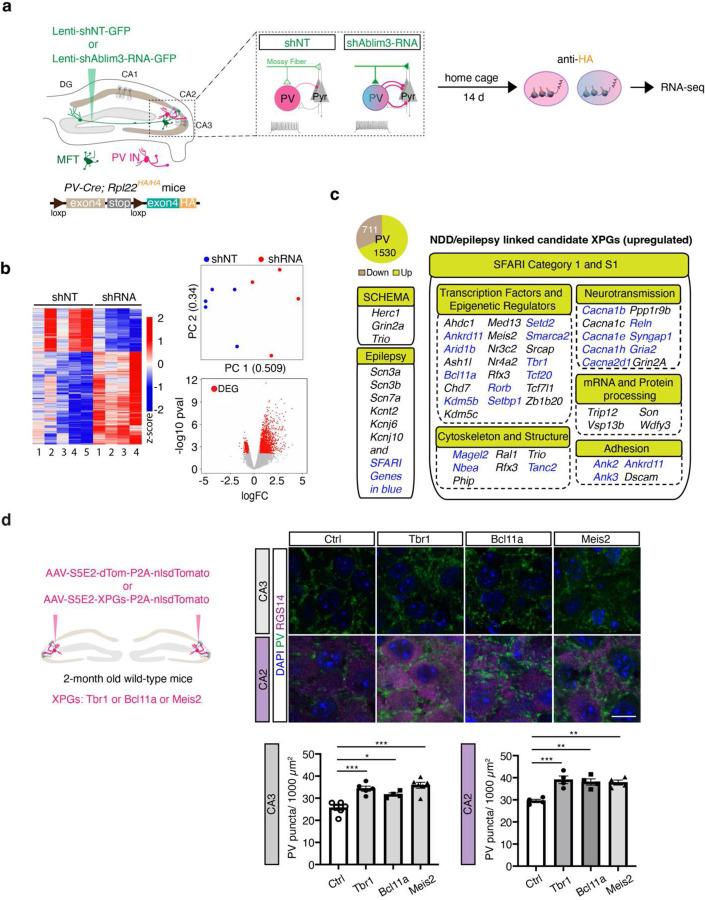
Screen for experience-dependent PV IN plasticity genes in adult CA2/CA3. **a**, Schematic of experimental workflow to biochemically isolate and sequence the translatome from naïve- and mossy fiber-triggered activated PV INs in adult *PV*^*Cre*^:*Rpl22HA*^*f/f*^ mice. Lentiviruses expressing *Ablim3* shRNA-GFP or non-targeting shRNA (shNT-GFP) were injected into the DG and two weeks later CA2/CA3 regions were microdissected. n=6 mice (3M and 3F) per sample, 5 samples/30 mice for shNT; 4 samples/24 mice for shRNA group. **b**, Left:Heatmap of expression values for differentially expressed genes, DEG, shown as normalized Z-scores relative to the average expression of a given gene across all samples. Right, top: Principal component analysis (PCA) plot of PV IN translatomes. The first two principal components are shown with the corresponding fractions of variance. Bottom, volcano plot showing statistical significance (-log10 P-value) vs. magnitude of change (log2 of fold change) of gene expression. DEGs are marked in red. **c**, Pie chart showing numbers of upregulated and downregulated XPGs. Upregulated XPGs linked to NDDs/epilepsies are highlighted. XPGs in blue font are implicated in epilepsies. **d**, Left: Experimental design. S5E2 enhancer AAV expressing control (dTomato) or XPGs (*Tbr1* or *Bcl11a* or *Meis2*) were injected into CA2/CA3 of 2-month-old wildtype mice 2 weeks prior to perfusion. Right: representative images and quantification of PV^+^ puncta density in CA3 (N = 6 mice for vector control; 5 mice for Tbr1; 4 mice for Bcl11a; 6 mice for Meis2) and CA2 (N=4 mice for each group). Scale bar, 10 μm. *p < 0.05; **p < 0.01; ***p < 0.001 using one-way ANOVA with Bonferroni post hoc test. All experiments are performed in male and female mice, and all data are displayed as mean ± SEM.

To identify candidate regulators of experience-dependent PV IN plasticity genes or “XPGs” in CA3/CA2 PV INs, we designed an in vivo functional screen in adult mice to isolate translated mRNAs from CA3/CA2 PV INs that receive increased mossy fiber inputs, a trigger for experience-dependent PV IN plasticity (Fig. 1a). To this end, we virally downregulated a molecular brake of mossy fiber filopodia-PV IN connectivity, Ablim3 (Non-target shRNA (shNT) vs. shAblim3-RNA), in DG mossy fibers^9–11^, to reproduce the effects of experience on mossy fiber filopodial inputs onto PV INs^9–11^. We used *PV-Cre; Rpl22*^*HA/HA*^ mice^32^, in which expression of the hemagglutinin (HA) epitope-tagged ribosomal protein L22 (Rpl22) is restricted to PV INs ^10^. Following optimization of signal-to-noise for mRNA isolation from this extremely sparse population of PV INs ^33^(Extended Data Fig. 1 a–b), we compared mRNAs biochemically isolated from genetically tagged PV INs in the naïve state versus an experience-induced activated state in CA3/CA2. Using this approach we identified differentially expressed candidate XPGs associated with the PV IN activated state (Fig.1b–c, Supplementary Table 1). Significantly upregulated genes included 67 Category 1, ASD SFARI genes (https://gene.sfari.org/)^34^, and 3 genes causally implicated in schizophrenia through exome sequencing via Schizophrenia Exome Sequencing Meta-analysis (SCHEMA)^35^. In sharp contrast, we identified only 3 category 1, syndromic ASD, genes in the list of significantly downregulated XPGs (Supplementary Table 1). Therefore, we focused on upregulated XPGs in this study. 18 out of 67 ASD genes encode histone methyltransferase, histone demethylases, ATP-dependent chromatin remodeling factors, transcription factors and co-activators whose functions in PV INs are poorly defined (Supplementary Table 1). Gene set enrichment analysis of all genes upregulated in experience-induced PV IN activated state suggests a role for these XPGs in synaptic physiology, structural remodeling and presynaptic terminal specialization (Extended Data Fig. 1c). Half of the ASD-linked upregulated XPGs are implicated in epilepsies, consistent with the high incidence of seizures in ASD individuals (Fig.1c)(https://gene.sfari.org/). Since ASD and SCZ individuals primarily carry loss-of-function mutations in these genes, the transcripts of which are upregulated in PV INs in response to increased mossy fiber inputs, our findings suggest that loss of PV IN experience-dependent plasticity is a convergent mechanism for different NDDs including ASD, epilepsies and schizophrenia.

After validating upregulation of select candidate ASD-linked XPGs (*Meis2, Bcl11a, Tbr1*) (https://gene.sfari.org/) and the SCHEMA gene, *Herc1*^35^, in activated CA3/CA2 PV INs by qPCR (Extended Data Fig. 1d), we asked whether boosting expression of these XPGs in PV INs using PV IN enhancer “S5E2” driven AAV viral vectors^36^ promotes formation of PV IN-CA3/CA2 synapses in adult mice. Viral upregulation of *Tbr1*, *Bcl11a*, *Meis2* or *Herc1* resulted in an increase in PV IN synapses in CA3 and CA2 pyramidal neurons (Fig.1d, Extended Data Fig. 1e). *Meis2* is expressed at negligibly low levels in a subpopulation of hippocampal PV INs in the hippocampus^37^ where its function is not known. MEIS2 is a homeodomain containing transcription factor that functions as a cellular context-dependent co-activator to regulate developmental specification of GABAergic projection neurons ^21,38^ and maintenance of intrinsic excitability and terminal arborization in postmitotic mechanosensitive sensory neurons^39^. Based on these observations we prioritized *Meis2* as a candidate XPG to restore experience-dependent PV IN plasticity in NDDs. Since many NDDs including ASD are characterized by a plethora of developmental deficits, intellectual disability, seizures and impaired social cognition, we selected *Cntnap2* KO (*Cntnap2*^*−/−*^) mice to test our hypothesis. Specifically, *Cntnap2*^*−/−*^mice exhibit an array of developmental deficits including loss of hippocampal and cortical PV INs, social and spatial cognitive impairments and seizures by around 4–6 months of age^40–43^.

## *Cntnap2* KO mice exhibit loss of social experience-dependent induction of PV IN synapses and *Meis2* upregulation in CA3/CA2 PV INs

**Fig. 2.**
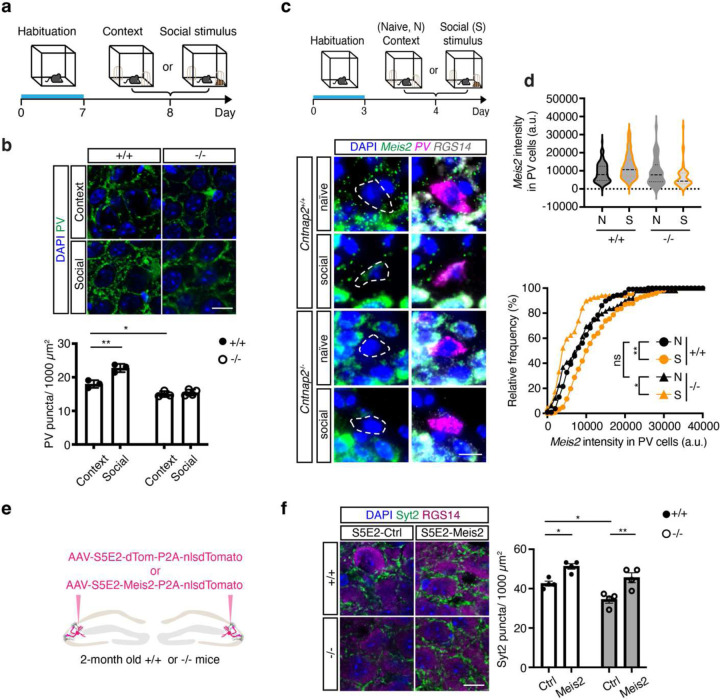
Reduced social experience-dependent induction of PV IN synapses and *Meis2* upregulation in CA3/CA2 PV INs of *Cntnap2* KO mice. **a**, Experimental design. Following habituation to a context, mice were exposed to a habituated context or a novel mouse for 10 min. **b**, Representative images and quantification of PV IN^+^ puncta (green, *N* = 3 mice for *Cntnap2*^*+/+*^, context and social groups; *N* = 3 mice for *Cntnap2*^*−/−*^, context group; *N* = 4 mice for *Cntnap2*^*−/−*^, social group) in CA3. *p < 0.05; **p < 0.01 using two-way ANOVA with Bonferroni post hoc test. Scale bar, 10 μm. **c**, Experimental design. Following habituation to a context, mice were exposed to a habituated context or a novel mouse for 10 min. Representative images of *Meis2* expression (green) in PV INs in *RGS14*^+^ labeling CA2 subfield. **d**, Top, quantification of *Meis2* expression (N = 3 mice for each group; for total PV INs, n= 107 for *Cntnap2*^*+/+*^, naïve group; n= 122 for *Cntnap2*^*+/+*^, social group; n= 63 for *Cntnap2*^*−/−*^, naïve group; n= 69 for *Cntnap2*^*−/−*^, social group). Bottom, cumulative probability plots of *Meis2* expression in PV INs. Kolmogorov-Smirnov test, *p < 0.05; **p < 0.01. **e**,**f**, 2-month old *Cntnap2*^*+/+*^ and ^*−/−*^ mice were injected with rAAV-S5E2-dTomato (Ctrl) or rAAV-S5E2-Meis2-nlsdTomato (Meis2) into CA2/CA3 2 weeks prior to quantification of Syt2^+^ puncta density in CA2 (N = 4 mice for each group). Scale bar, 10 μm. *p < 0.05; **p < 0.01 using two -way ANOVA with Bonferroni post hoc test. All experiments are performed in male and female mice, and all data are displayed as mean ± SEM.

We asked whether CA3/CA2 PV INs in adult (2 months old)*Cntnap2* KO (*Cntnap2*^*−/−*^) mice retain competence to increase their perisomatic synapses in response to social experience. Our analysis revealed that *Cntnap2 KO* mice had fewer PV IN perisomatic synapses (PV puncta) in CA3 as compared to wild-type littermates (*Cntnap2*^*+/+*^)(Fig. 2a–b). Additionally, exposure to a social stimulus following habituation to a context resulted in an increase in PV IN perisomatic synapses in *Cntnap2*^*+/+*^ mice but not in *Cntnap2*^*−/−*^ littermates (Fig. 2a–b). Next, we asked whether *Meis2* expression levels in CA3/CA2 PV INs in adult *Cntnap2*^*−/−*^ mice and *Cntnap2*^*+/+*^ littermates change in response to social experience. Multiplex fluorescent *in situ* hybridization for *Meis2*, *PV* and *RGS14* in hippocampal sections obtained from mice habituated to a context (social experience naïve) and mice exposed to a social stimulus in the habituated context revealed a social experience-dependent increase in *Meis2* transcripts (*Meis2* intensity per PV IN and proportion of PV INs that express *Meis2*) in *Cntnap2*^*+/+*^ mice but not in *Cntnap2*^*−/−*^ mice (Fig. 2c–d).

## *Meis2*-dependent restoration of CA3/CA2 PV IN synapses, inhibitory and excitatory synaptic transmission and PV IN synaptic plasticity in adult *Cntnap2* KO mice

**Extended Data Fig. 2.**
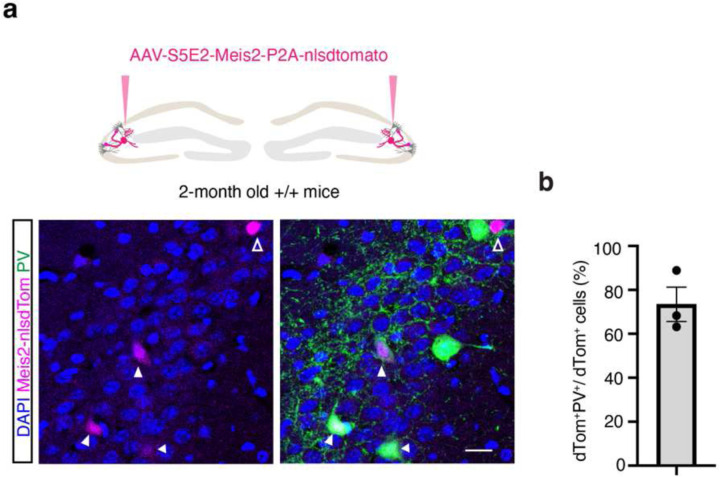
Specificity of AAV-S5E2-Meis2-nlsdTomato targeting PV INs. **a**, 2-month-old wildtype mice were injected with rAAV-S5E2-Meis2-nlsdTomato. Representative images of Meis2-dTom overlapping with PV^+^ cells in CA2/CA3 subfield. Scale bar, 20 μm. **b**, Percentage of dTom^+^PV^+^/total dTom^+^ cells (N = 3 mice). All data are displayed as mean ± SEM.

**Extended Data Fig. 3.**
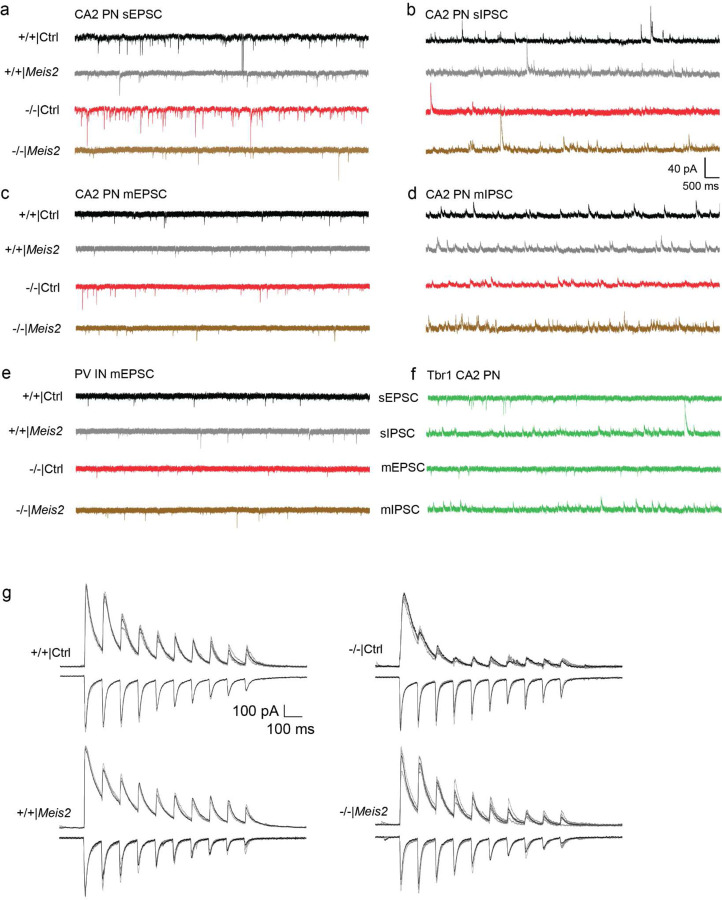
Representative traces from ex vivo slice recordings. **a-b**, Representative traces of sEPSC and sIPSC recordings onto CA2 PNs. **c-d**, Representative traces of mEPSC and mIPSC recordings onto CA2 PNs. **e**, Representative traces of mEPSC onto PV INs. **f**, Representative traces of sEPSC, sIPSC, mEPSC, and mIPSC onto CA2 PNs from *Cntnap2*^*+/+*^ mice injected with AAV-S5E2-Tbr1-P2A-nlsTomato along the stratum lucidum. **g**, Representative traces depicting EPSC (downward deflections, voltage clamp = −70 mV) and IPSC (upward deflections, voltage clamp = 0 mV) elicited by 10 burst optic stimulation train.

**Fig. 3.**
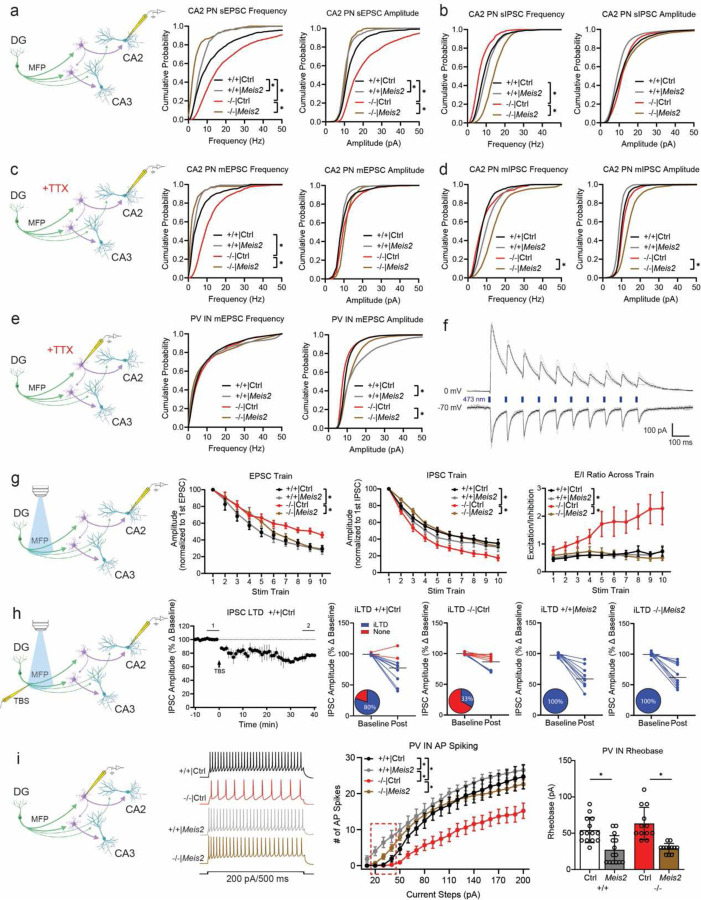
*Meis2*-dependent restoration of excitatory and inhibitory transmission upon CA2 PNs, PV IN excitability, and inhibitory-dependent synaptic plasticity in *Cntnap2*^*−/−*^ mice. **a**, Schematic depicting mossy fiber pathway and whole-cell voltage-clamp recording of spontaneous excitatory postsynaptic current (sEPSC) from CA2 pyramidal neurons (PN). *Cntnap2*^*−/−*^ or ^+/+^ mice were injected with rAAV-S5E2-Meis2-P2A-nlsdTomato or rAAV-S5E2-dTomato (Ctrl) virus along the stratum lucidum mossy fiber pathway adjacent to CA2/CA3a pyramidal cell layer of the dorsal hippocampus. Cumulative probability plots of sEPSC frequency and amplitude from CA2 PNs. Kolmogorov-Smirnov test, *p < 0.05, n=8–9 cells, 1–3 cells per mouse, 5–9 mice per group. **b**, Cumulative probability plots of spontaneous inhibitory postsynaptic current (sIPSC) frequency and amplitude from CA2 PNs. Kolmogorov-Smirnov test, *p < 0.05, n=8–9 cells, 1–3 cells per mouse, 5–9 mice per group. **c**, Schematic depicting mossy fiber pathway and whole-cell voltage-clamp recording of miniature excitatory postsynaptic current (mEPSC) from CA2 PNs. Bath application of tetrodotoxin (TTX, 1 µM) was done to block voltage-gated sodium channels. Cumulative probability plots of mEPSC frequency and amplitude from CA2 PNs. Kolmogorov-Smirnov test, *p < 0.05, n=8–9 cells, 1–3 cells per mouse, 5–9 mice per group. **d**, Cumulative probability plots of miniature inhibitory postsynaptic current (sIPSC) frequency and amplitude from CA2 PNs. Kolmogorov-Smirnov test, *p < 0.05, n=8–9 cells, 1–3 cells per mouse, 5–9 mice per group. **e**, Schematic depicting whole-cell voltage-clamp recording of mEPSC from PV INs along the stratum lucidum of the dorsal hippocampus. Cumulative probability plots of mEPSC frequency and amplitude from PV INs. Kolmogorov-Smirnov test, *p < 0.05, n=9–15 cells, 2–4 cells per mouse, 3–6 mice per group. **f**, Representative traces depicting 10 burst optic stimulation train (473 nm blue light, 1 ms pulse duration, 100 ms inter-stim interval repeated 5 times with 20 sec between trains) delivered above the mossy fiber pathway proximal to the hilus of the DG. Optically evoked EPSCs were recorded by voltage-clamp at −70 mV and IPSCs by voltage-clamp at 0 mV. **g**, Schematic depicting mossy fiber driven optically evoked EPSC and IPSC recorded from CA2 PNs. Mice were injected with pAAV5-CamKIIa-hChR2-eYFP into the dorsal dentate gyrus. Line graphs depict EPSC, IPSC, and excitation/inhibition ratio across optic evoked stimulation train. Graphs were analyzed with Two-way RM ANOVA with Tukey posthoc, *p < 0.05, n=10–12 cells, 1–2 cells per mouse, 6–9 mice per group. **h**, Schematic depicting mossy fiber pathway and whole-cell voltage-clamp onto CA2 PNs. A bipolar stimulating electrode contained in a patch pipette filled with ACSF was placed along the hilus of the DG along the mossy fiber pathway. Plasticity was induced by electrically evoked theta-burst stimulation (TBS), which consisted of bursts of 4 pulses at 100 Hz, interburst interval was 200 ms, and repeated 4 times at 10 sec intervals. IPSCs or EPSCs were optically evoked. Line graph depicts long-term depression of optically evoked IPSC (iLTD) in a *Cntnap2*^+/+^ mouse. Significance (*p* < 0.05) was assessed by unpaired *t* test between baseline (1^st^ bar at −5 to 0 min) and 35 minutes post theta-burst stimulation (2^nd^ bar at 35 to 40 min). Two group iLTD graphs depict IPSC amplitude between baseline and post. Blue lines depict cells that depressed and red lines depict no statistical difference between baseline and post stimulation. Pie charts display the percent of total recorded neurons that depressed. Recordings were from n=9–10 cells, 1 cell per slice, 1–2 slices per mouse, 8–10 mice per group. **i**, Schematic depicting whole-cell current-clamp onto PV INs along the stratum lucidum adjacent to CA2/CA3a pyramidal cell layer of the dorsal hippocampus. Representative current traces from each group depict action potential response to current steps (200 pA, 500 ms). The line graph depicts the number of action potential (AP) spike responses to incremental current steps. Two-way RM ANOVA with Tukey posthoc, *p < 0.05, n=9–12 cells, 2–3 cells per mouse, 4–5 mice per group. The bar graph depicts the rheobase, the minimum current required to induce an action potential. Two-way ANOVA with Uncorrected Fisher’s LSD posthoc, *p < 0.05, n=12–14 cells, 2–3 cells per mouse, 4–5 mice per group.

To acutely boost *Meis2* levels in CA3/CA2 PV INs, we generated a S5E2 PV IN enhancer driven Meis2-P2A-nlsdTomato AAV that preferentially targets expression of *Meis2* in PV INs including basket cells and axo-axonic cells in CA3/CA2 (Extended Data Fig. 2a–b). Virally boosting *Meis2* in CA3/CA2 of adult *Cntnap2*^*−/−*^ mice was sufficient to restore PV IN perisomatic synapses in CA2 as quantified using synaptotagmin 2 immunohistochemistry^8^(Fig. 2e–f). We next determined if anatomical restoration of PV IN synapses in CA2 of adult *Cntnap2*^*−/−*^ mice was accompanied by changes in PV IN properties, physiology and synaptic transmission in the DG-CA2 circuit. We targeted expression of *Meis2* or *dTomato* in PV INs along the stratum lucidum mossy fiber pathway adjacent to CA2/CA3a pyramidal cell layer of the dorsal hippocampus in adult *Cntnap2*^*−/−*^ mice or ^+/+^ littermates using AAV-S5E2-Meis2-P2A-nlsdTomato or AAV-S5E2-dTom-P2A-nlsdTomato control viruses. E*x vivo* whole-cell patch-clamp recordings from PV INs and CA2 pyramidal neurons (PN) revealed that *Cntnap2*^*−/−*^ mice exhibit increased spontaneous excitatory postsynaptic current (sEPSC) in CA2 PNs compared to *Cntnap2*^+/+^ mice and boosting *Meis2* in PV INs reduced sEPSC frequency and amplitude in *Cntnap2*^*−/−*^ and *Cntnap2*^*+/+*^ mice (Fig.3a, Extended Data Fig. 3a). Spontaneous inhibitory postsynaptic current (sIPSC) frequency in CA2 PNs from *Cntnap2*^*−/−*^ was reduced compared to *Cntnap2*^+/+^ littermates, and boosting *Meis2* in PV INs reversed this reduction in *Cntnap2*^*−/−*^ mice (Fig.3b, Extended Data Fig. 3b). To assess action potential-independent spontaneous vesicle release, we recorded miniature excitatory and inhibitory postsynaptic current (mEPSC/mIPSCs) from CA2 PNs. We found that *Cntnap2*^*−/−*^ mice have higher mEPSC frequency than *Cntnap2*^*+/+*^ littermates, and boosting *Meis2* in PV INs reversed this increase (Fig.3c, Extended Data Fig. 3c). There was no difference in the frequency or amplitude of mIPSCs from CA2 PN between *Cntnap2*^*−/−*^ and *Cntnap2*^*+/+*^ mice, however boosting *Meis2* in PV INs increased mIPSC frequency and amplitude in CA2 PNs of *Cntnap2*^*−/−*^ mice (Fig.3d, Extended Data Fig. 3d). We assessed mEPSC in PV INs and found that boosting *Meis2* in these interneurons increased mEPSC amplitude in both *Cntnap2*^*−/−*^ and *Cntnap2*^+/+^ mice (Fig.3e, Extended Data Fig. 3e).

**Extended Data Fig. 4.**
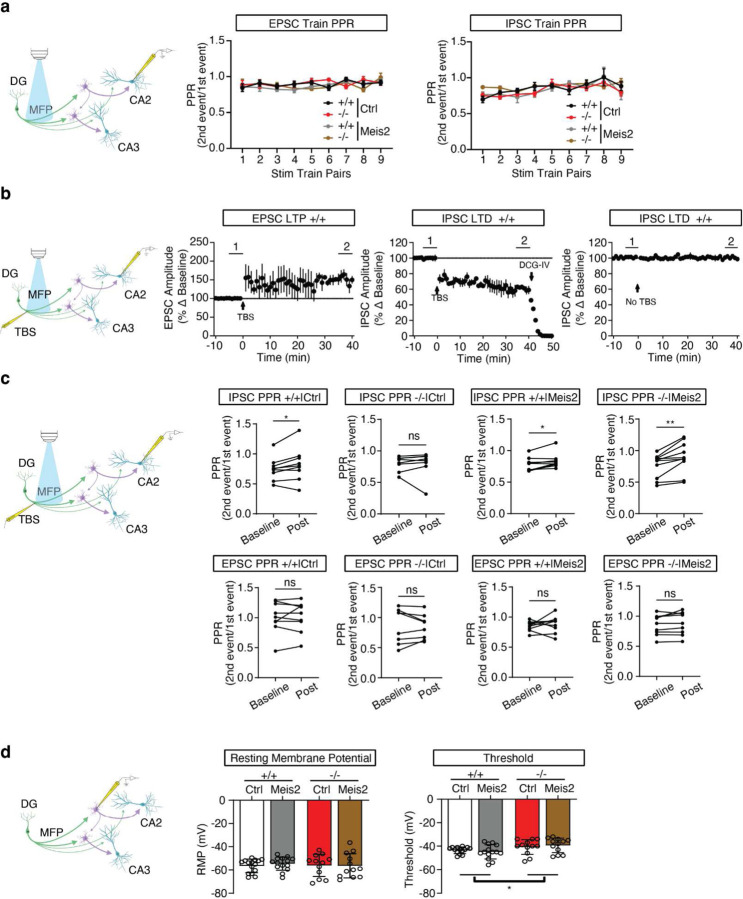
Supporting data for electrophysiological characterization of viral-restoration of *Meis2* upregulation in CA3/CA2 PV INs in *Cntnap2*^*−/−*^ or ^+/+^ mice. **a**, Schematic depicting mossy fiber driven optically evoked EPSC and IPSC recorded from CA2 PNs. Mice were injected with pAAV5-CamKIIa-hChR2-eYFP into the dorsal DG. Line graphs depict paired pulse ratios (2^nd^ event amplitude/1^st^ event amplitude) across the 10-burst optic stimulation train. Two-way RM ANOVA, *p < 0.05, n=8–9 cells, 1–3 cells per mouse, 4–7 mice per group. **b**, Schematic depicting mossy fiber pathway and whole-cell voltage-clamp onto CA2 PNs. A bipolar stimulating electrode contained in a patch pipette filled with ACSF was placed along the hilus of the DG along the mossy fiber pathway. Plasticity was induced by electrically evoked theta-burst stimulation (TBS) which consisted of bursts of 4 pulses at 100 Hz, interburst interval was 200 ms, and repeated 4 times at 10 sec intervals. IPSCs or EPSCs were optically evoked. Left line graph depicts long-term potentiation (LTP) of optically evoked EPSC in a ^+/+^ mouse. Significance (p < 0.05) was assessed by unpaired *t* test between baseline (1^st^ bar at −5 to 0 min) and 35 minutes post TBS (2^nd^ bar at 35 to 40 min). The middle line graph depicts long-term depression of IPSC (iLTD) followed by bath application of DCG-IV, group II metabotropic glutamate receptor agonist which suppresses synaptic transmission at mossy fiber pathway. The right line graph depicts optically evoked IPSC without TBS plasticity induction. **c**, Schematic depicting mossy fiber pathway and whole-cell voltage-clamp onto CA2 PNs. Electrically evoked TBS was delivered along the mossy fiber pathway. Paired pulse ratios (PPR) of the IPSC and EPSC were recorded from CA2 PNs at baseline (−5 to 0 min) and post TBS (35 to 40 min). Data was analyzed with paired *t* tests, *p < 0.05, n=9–10 cells, 1 cell per slice, 1–2 slices per mouse, 8–10 mice per group. **d**, Schematic depicting whole-cell current-clamp onto PV INs along the stratum lucidum adjacent to CA2/CA3a pyramidal cell layer of the dorsal hippocampus. *Cntnap2*^*−/−*^ or ^+/+^ mice were injected with AAV-S5E2-Meis2-P2A-nlsdTomato or AAV-P2A-nlsdTomato control virus along the stratum lucidum mossy fiber pathway adjacent to CA2/CA3a pyramidal cell layer of the dorsal hippocampus. Bar graphs depict the resting membrane potential (RMP) and threshold of PV INs. Two-way ANOVA with Uncorrected Fisher’s LSD posthoc, *p < 0.05, n=12–14 cells, 2–3 cells per mouse, 4–5 mice per group.

To assess mossy fiber driven evoked current onto CA2 PNs, we injected pAAV5-CamKIIa-hChR2-eYFP into the dorsal DG and AAV-S5E2 driven *Meis2* or *dTtomato* expressing viruses into CA3/CA2 and elicited optically evoked stimulation trains targeting the mossy fiber pathway (a train of 10 optic stimulations, 1 ms pulse duration, 100 ms inter-stim interval repeated 5 times with 20 sec between trains) (Fig.3f). We found that *Cntnap2*^*−/−*^ mice exhibit higher amplitude optically-evoked EPSCs and lower amplitude IPSCs across the stim train compared to *Cntnap2*^+/+^ mice. Boosting *Meis2* in PV INs attenuated evoked EPSC and increased evoked IPSC amplitude to responses comparable to those observed in *Cntnap2*^*+/+*^ mice (Fig.3g, Extended Data Fig. 3g). Compared to all other groups, *Cntnap2*^*−/−*^ mice exhibit an overall higher excitation to inhibition ratio (Fig.3g). We observed no differences in vesicle release probability across the stimulation train, indicating the stimulation frequency does not induce short-term plasticity (Extended Data Fig. 4a). These results suggest *Cntnap2*^*−/−*^ mice have higher frequency of excitatory transmission and reduced inhibition onto CA2 PNs and that boosting *Meis2* in PV INs reverses these developmental phenotypes. The reduction in excitatory synaptic transmission in CA2 may arise in response to sustained increases in MEIS2-dependent inhibition of pyramidal neurons.

Next, we asked whether boosting *Meis2* in PV INs influences mossy fiber driven, inhibitory-dependent synaptic plasticity (iLTD) which is thought to facilitate routing of information ^44^. To elicit inhibitory-dependent plasticity, we stimulated the mossy fiber pathway by electrical theta burst stimulation (TBS) and recorded from CA2 PNs^44^. TBS along the mossy fiber pathway induces long-term depression of the IPSC (iLTD) and long-term potentiation (LTP) of the EPSC upon CA2 PNs (Fig.3h, Extended Data Fig. 4b). We found that *Cntnap2*^*−/−*^ mice exhibit reduced occurrence of iLTD compared to *Cntnap2*^+/+^ littermates, and boosting *Meis2* in PV INs restored the occurrence of iLTD in both *Cntnap2*^*−/−*^ and *Cntnap2* ^+/+^ mice (Fig.3h). We assessed vesicle release probability of IPSC and EPSC at baseline (5 min before TBS) and post iLTD (between 35 to 40 min post TBS). We found that the vesicle release probability of IPSC was reduced from baseline to post iLTD in all other groups except *Cntnap2*^*−/−*^ mice. There were no changes in vesicle release probability in the EPSC across all groups (Extended Data Fig. 4c). This suggests that mossy fiber driven iLTD and subsequent LTP of the EPSC upon CA2 PNs is a presynaptic mechanism in inhibitory synapses.

Next, we assessed how boosting *Meis2* in PV INs influences PV IN passive membrane properties in the four groups. Current injections in PV INs revealed reduced excitability in *Cntnap2*^*−/−*^ mice and boosting *Meis2* in these interneurons restored excitability and reduced rheobase in both *Cntnap2*^*−/−*^ and ^+/+^ mice (Fig. 3i). We found no differences in the resting membrane potential in any of the groups, however PV INs from *Cntnap2*^*−/−*^ mice exhibit a more depolarized threshold. Interestingly, boosting *Meis2* did not affect the threshold in *Cntnap2*^*−/−*^ or in *Cntnap2*^+/+^ mice, suggesting that the depolarized threshold arises from a Meis2-independent compensatory mechanism (Extended Data Fig. 4d).

**Extended Data Fig. 5.**
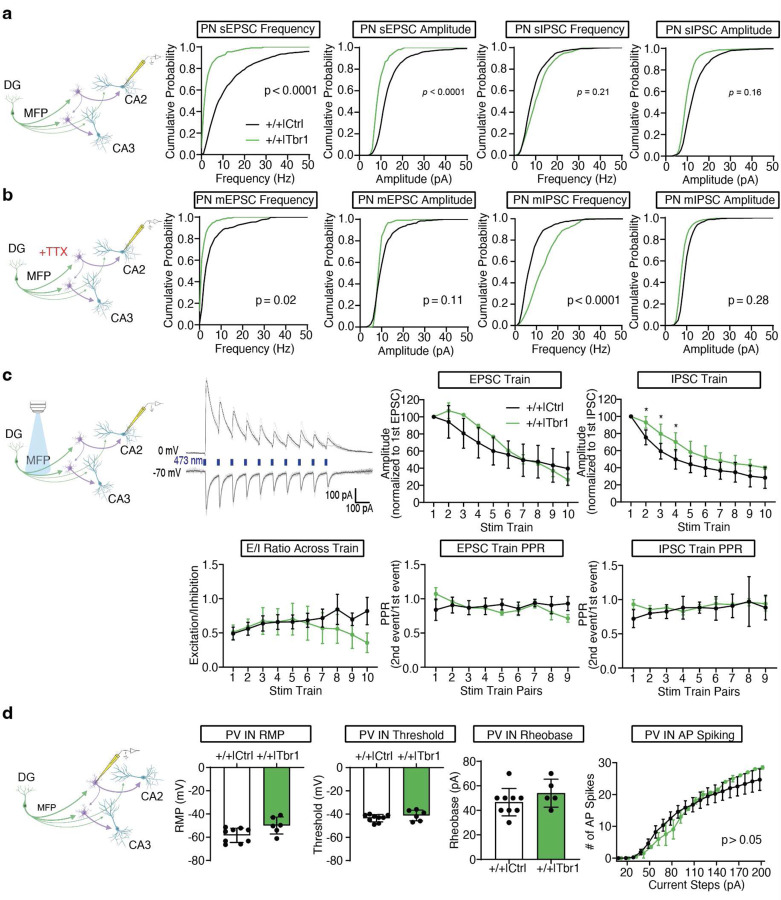
Boosting *Tbr1* in CA3/CA2 PV INs reduces excitation and increases inhibitory synaptic transmission in *Cntnap2*^*+/+*^ mice. **a**, Schematic depicting mossy fiber pathway and whole-cell voltage-clamp recording of spontaneous excitatory and inhibitory postsynaptic current (sEPSC/sIPSC) from CA2 pyramidal neurons (PN). *Cntnap2*^*+/+*^ mice were injected with AAV-S5E2-Tbr1-P2A-nlsdTomato or AAV-P2A-nlsdTomato control virus along the stratum lucidum mossy fiber pathway adjacent to CA2/CA3a pyramidal cell layer of the dorsal hippocampus. Cumulative probability plots of sEPSC and sIPSC frequency and amplitude from CA2 PNs. Kolmogorov-Smirnov test, n=3–7 cells, 1–2 cells per mouse, 3–7 mice per group. ^+/+^ data from Figure 3 and Figure 3S were used as control comparison group in these experiments. **b**, Schematic depicting mossy fiber pathway and whole-cell voltage-clamp recording of miniature excitatory and inhibitory postsynaptic current (mEPSC/mIPSC) from CA2 PNs. Bath application of tetrodotoxin (TTX, 1 µM) was done to block voltage-gated sodium channels. Cumulative probability plots of mEPSC and mIPSC frequency and amplitude from CA2 PNs. Kolmogorov-Smirnov test, n=4–7 cells, 1–2 cells per mouse, 4–7 mice per group. **c**, Schematic depicting mossy fiber driven optically evoked EPSC and IPSC recorded from CA2 PNs. Mice were injected with pAAV5-CamKIIa-hChR2-eYFP into the dorsal dentate gyrus. Representative traces depicting 10 burst optic stimulation train (473 nm blue light, 1 ms pulse duration, 100 ms inter-stim interval repeated 5 times with 20 sec between trains) delivered above the mossy fiber pathway proximal to the hilus of the DG. Optically evoked EPSCs were recorded by voltage-clamp at −70 mV and IPSCs by voltage-clamp at 0 mV. Line graphs depict EPSC, IPSC, excitation/inhibition ratio and paired pulse ratio (PPR) across optic evoked stimulation train. Graphs were analyzed with Two-way RM ANOVA with Uncorrected Fisher’s LSD posthoc, *p < 0.05, n=2–8 cells, 1–2 cells per mouse, 2–5 mice per group. **d**, Schematic depicting whole-cell current-clamp onto PV INs along the stratum lucidum adjacent to CA2/CA3a pyramidal cell layer of the dorsal hippocampus. *Cntnap2*^*+/+*^ mice were injected with AAV-S5E2-Tbr1-P2A-nlsdTomato or AAV-P2A-nlsdTomato control virus along the stratum lucidum mossy fiber pathway adjacent to CA2/CA3a pyramidal cell layer of the dorsal hippocampus. Bar graphs depict PV IN resting membrane potential (RMP), threshold, and rheobase. Bar graphs were analyzed with unpaired *t* test, *p < 0.05, n=5–9 cells, 2–3 cells per mouse, 3–4 mice per group. The line graph depicts the number of action potential (AP) spike responses to incremental current steps. Two-way RM ANOVA with Tukey posthoc, *p < 0.05, n=2–9 cells, 2–3 cells per mouse, 2–4 mice per group.

To determine if another XPG, the T-brain-1 (*Tbr1*), a T-box transcription factor, and *Meis2* have overlapping or distinct roles in regulating PV IN physiology, we injected AAV-S5E2-*Tbr1*-*P2A-nlsdTomato* along the stratum lucidum adjacent to CA2/CA3a pyramidal cell layer in *Cntnap2*^+/+^ mice and performed recordings from PV INs and CA2 pyramidal neurons (Extended Data Fig. 5, Extended Data Fig. 3f). We found that boosting *Tbr1* in PV INs results in reduced sEPSC frequency and amplitude, reduced mEPSC frequency, and increased mIPSC frequency upon CA2 PNs (Extended Data Fig. 5a–b, Extended Data Fig. 3f). Mossy fiber driven evoked current onto CA2 PNs with a 10 burst optic stimulation train revealed an increase in IPSC amplitude, however there were no significant differences in excitation/inhibition ratio compared to *Cntnap2*^+/+^ mice (Extended Data Fig. 5c). Boosting *Tbr1* in PV INs did not result in any differences in intrinsic excitability and passive membrane properties from PV INs (Extended Data Fig. 5d).

Together, these observations demonstrate that boosting *Meis2* expression in CA3/CA2 PV INs of adult *Cntnap2*^*−/−*^ mice reverses developmental deficits in PV IN intrinsic excitability, input-output synaptic connectivity, excitatory and inhibitory synaptic transmission and inhibitory-dependent synaptic plasticity. Additionally, our analysis of *Meis2* and *Tbr1* suggests that these two XPGs independently and differentially contribute to experience-dependent PV IN plasticity.

## *Meis2*-dependent rescue of cognition in adult *Cntnap2* KO mice

**Extended Data Fig. 6.**
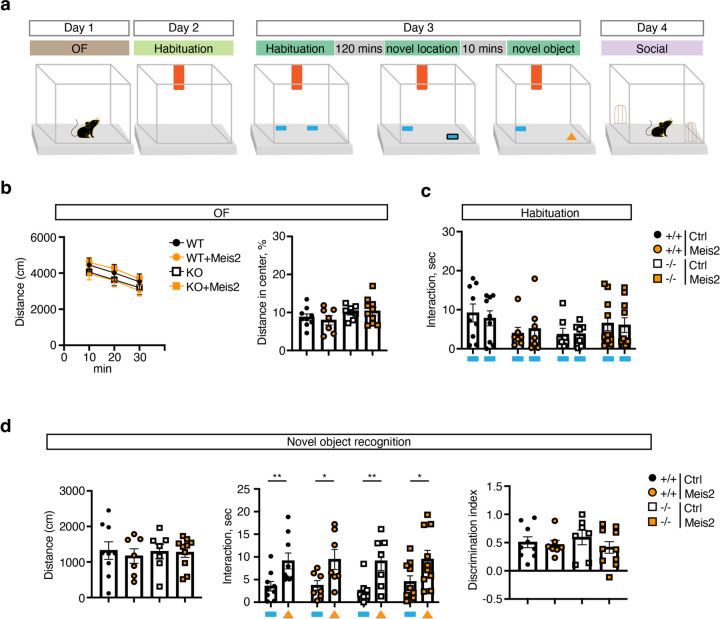
Boosting *Meis2* expression in CA2/CA3 PV INs does not affect locomotion and novel object recognition in *Cntnap2* KO mice. **a**, Schematic of behavior testing schedule. Day 1, OF, open-field task; Day 2, habituation to visual cue; Day 3, NOL, novel object location and NOR, novel object recognition; Day 4, Social cognition task. **b**, OF, quantification of total distance traveled, percentage of distance traveled across the center arena, percentage time in center. **c**, Quantification of the time spent sniffing two identical objects (seconds) during habituation on Day 3. **d**, NOR, novel object recognition, mouse was exposed to 2 objects over three sessions (5 minutes each session, object locations counterbalanced). Time spent sniffing the objects was quantified. These experiments were performed in male and female mice. All data are displayed as mean ± SEM and analyzed using two-way ANOVA with Bonferroni post hoc test.

**Extended Data Fig. 7.**
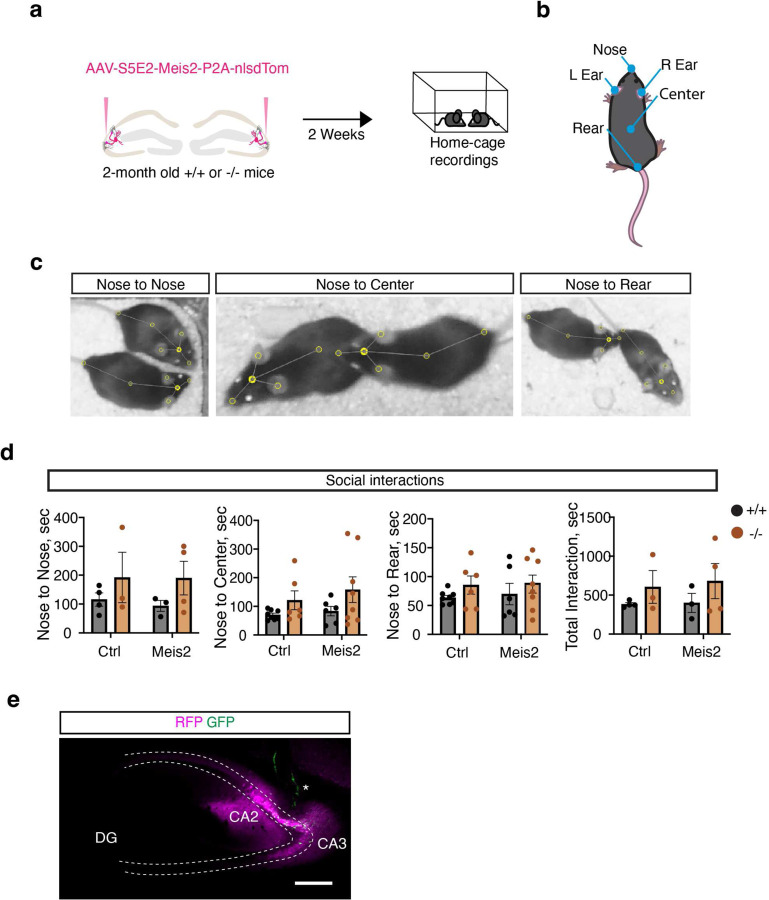
Boosting *Meis2* expression in CA2/CA3 PV INs does not affect group social behavior of *Cntnap2* KO and wild-type littermates in homecage. **a**, Pairs of 2-month-old *Cntnap2*^*+/+*^ or ^−/−^ mice were injected with either AAV-S5E2-Meis2-nlsdTom or AAV-S5E2-dTom constructs. Following a 2-week incubation period, mice were recorded for 1 hour in their home cages during their night cycle to monitor social interactions. **b**, Schematic showing node locations (nose, R ear, L ear, center, and rear) used for pose estimation via SLEAP. **c**, Representative node placements during three types of social interaction: nose-to-nose, nose-to-center, and nose-to-rear. **d**, Quantification of social interaction data across conditions (3–4 cages per condition, 2 mice per cage), comparing *Cntnap2*^+/+^ and ^−/−^ groups injected with either tdTomato or Meis2 constructs. Nose to nose, nose to center, nose to rear and total Interaction were analyzed (sec). These experiments were performed in male and female mice. **e**, Representative image of Cal-Light viruses expression and implant in CA2/CA3 subfield (indicated by asterisk). Scale bar, 250 μm. All data are displayed as mean ± SEM and analyzed using two-way ANOVA with Bonferroni post hoc test.

**Fig. 4.**
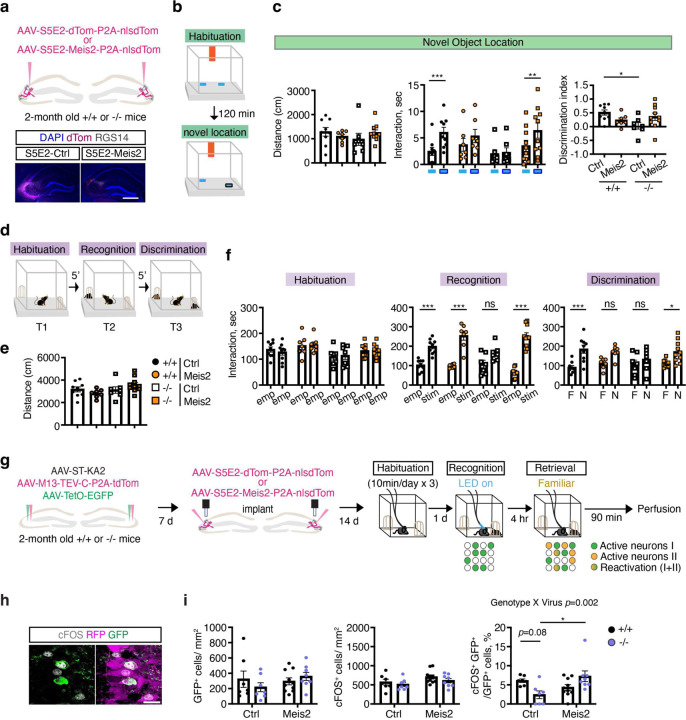
*Meis2*-dependent rescue of cognition in adult *Cntnap2* KO mice. **a**, 2-month old *Cntnap2*^*+/+*^ and ^*−/−*^ mice were injected with rAAV-S5E2-dTomato (Ctrl) or rAAV-S5E2-Meis2-nlsdTomato (Meis2, nls=nuclear localization signal) into CA2/CA3 2 weeks prior to behavioral testing (N = 9 for +/+ mice with Ctrl; 7 mice for +/+ with Meis2; 7 mice for −/− with Ctrl; 10 mice for −/− with Meis2). Representative images of Ctrl and Meis2 expression in CA2/CA3 subfield. Scale bar, 500 μm. **b**, Schematic of novel object location task (Extended Data Fig. 6 for behavioral testing schedule). **c**, Quantification of total distance travelled, and time spent investigating objects. **d**, Schematic of social cognition task depicting habituation trial T1, encoding/recognition trial, T2; and social memory discrimination trial, T3. **e**, Quantification of total distance travelled during T1 trial. **f**, Quantification of interaction time during habituation (T1), social recognition phase (T2) and social stimuli interaction time in the discrimination trial (T3) (N =11 mice per group). Emp, empty pencil cup; stim, stimulus mouse; F, familiar mouse; N, a new novel stimulus mouse. These experiments are performed in male and female mice. All data are displayed as mean ± SEM and analyzed using two-way ANOVA with Bonferroni post hoc test. ns, not significant; *p < 0.05; **p < 0.01; ***p < 0.001. **g**, Experimental workflow for tagging neurons active during social interaction and assessing reactivation of tagged neuronal ensemble. 2-month-old *Cntnap2*^+/+^ and ^−/−^ mice were injected with Cal-Light viruses (AAV-ST-KA2, AAV-M13-TEV-C-P2AtsTom, AAV-TetO-EGFP) into the CA2/3 region. One week later, mice were injected either AAV-S5E2-Meis2 (Meis2) or AAV-S5E2-dTomato (Ctrl) and were implanted with fiberoptic probes above CA2/3 14 days prior to behavioral testing. (N = 7 for +/+ and −/− mice with Ctrl; 10 mice for +/+ with Meis2; 8 mice for −/− with Meis2). **h**, Representative image of tagged neurons. Scale bar, 20 μm. **i**, Quantification of Cal-light tagged active neurons (GFP^+^), cFos^+^ neurons, and reactivation ratio (cFOS^+^ GFP^+^ /GFP^+^ cells) in *Cntnap2*^+/+^ and ^−/−^ mice injected with either Ctrl or Meis2 expressing viruses. These experiments are performed in male and female mice. All data are displayed as mean ± SEM and analyzed using two-way ANOVA with Tukey post hoc test. ns, not significant; *p < 0.05.

We next asked whether *Meis2*-dependent restoration of experience-dependent PV IN plasticity in CA3/CA2 PV INs of adult *Cntnap2*^*−/−*^ mice rescues spatial and social cognition impairments. Following viral upregulation of *Meis2* in PV INs, adult (2–3 months) *Cntnap2*^*+/+*^ and ^−/−^ mice were tested in the novel object location task that probes the capacity of the hippocampus to bind object and spatial information into conjunctive representations, or “contexts”(Fig. 4a–b). Consistent with a role for DG-CA3/CA2 circuitry in encoding configural relationships between objects and their locations within contexts, *Cntnap2*^*+/+*^ but not *Cntnap2*^*−/−*^ mice spent more time exploring the displaced object over the non-displaced object in the habituated context. Increasing *Meis2* in CA3/CA2 PV INs of *Cntnap2*^*−/−*^ mice rescued cognitive behavioral impairment in this task, matching behavior of *Cntnap2*^*+/+*^ littermates (Fig. 4c). In the social recognition memory paradigm, mice are habituated to a context in trial 1 (T1) to two empty inverted cups, encode a social stimulus in one cup in T2 (counter balanced, familiarization phase), and are then challenged to discriminate between the familiar social stimulus (from T2) and a new social stimulus in trial 3 (Fig. 4d). Trial T2 probes social recognition (often referred to as sociability) and is dependent on DG recruitment of feed-forward inhibition of CA2^9^. All four groups of mice equivalently explored the context and both empty cups in the habituation phase (Fig. 4e–f). However, *Cntnap2*^*+/+*^ but not *Cntnap2*^*−/−*^ mice, spent more time exploring the social stimulus over the empty cup in trial 2 and discriminated between the novel social stimulus and the familiar social stimulus in trial 3. Boosting *Meis2* in CA3/CA2 PV INs of *Cntnap2*^*−/−*^ mice reversed these impairments in social recognition and social discrimination (Fig. 4f). Unlike in *Cntnap2*^*−/−*^ mice, elevating *Meis2* levels in CA3/CA2 PV INs of *Cntnap2*^*+/+*^ mice impaired social and spatial cognition. Thus, when GABAergic inhibition is intact, increasing MEIS2 dosage does not enhance cognition (Fig. 4c, 4f). Analysis of this same cohort of mice in assays for anxiety-like behavior (open field), novel object recognition and homecage group social behavior using Social LEAP Estimates Animal Poses or SLEAP^45^ did not detect differences between these groups (Extended Data Fig. 6a–d, Extended Data Fig. 7a–d).

Increased MEIS2 levels in CA3/CA2 PV INs may rescue social recognition in *Cntnap2*^*−/−*^ by promoting specificity of neuronal ensembles encoding social stimuli. To test this hypothesis, we used a dual light and calcium-dependent neuronal activity tagging system, soma-targeted Cal-Light ^46^, to tag active neurons (with induction of GFP) during exploration of social stimulus in a habituated context and then quantify reactivation of the social stimulus-associated ensemble upon re-exposure to same social stimulus. Higher reactivation [% (cFOS+GFP+)/(GFP+ cells)] suggests increased specificity of the neuronal ensemble (Fig. 4g, Extended Data Fig. 7e). In both *Cntnap2*^*+/+*^ and *Cntnap2*^*−/−*^ mice, we detected equivalent numbers of active cells in recognition and retrieval phases, however, boosting *Meis2* in CA3/CA2 PV INs of *Cntnap2*^*−/−*^ mice significantly enhanced ensemble reactivation of the social stimulus-associated ensemble compared with control virus infected *Cntnap2*^*−/−*^ mice (Fig. 4h–i). Together, these observations demonstrate that boosting *Meis2* expression in CA3/CA2 PV INs of adult *Cntnap2*^*−/−*^ mice reverses developmental deficits in hippocampal dependent cognition and promotes ensemble specificity.

## *Meis2*-dependent suppression of seizures in *Cntnap2* KO mice

**Extended Data Fig. 8.**
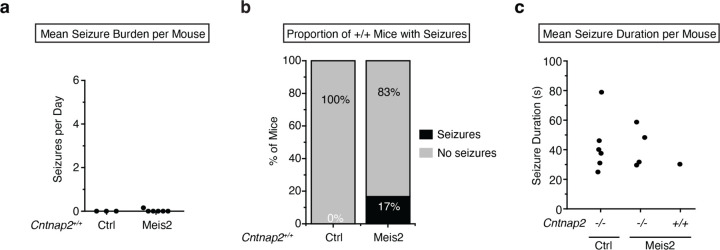
Analysis of seizure frequency in *Cntnap2*^*+/+*^ mice and seizure duration for all groups. **A**, ECoG recordings for 2 weeks revealed no seizures in *Cntnap2*^*+/+*^ mice treated with AAV-S5E2-dTom and 2 seizures in a single *Cntnap2*^*+/+*^ mouse treated with AAV-S5E2-Meis2. **b.** The proportion of *Cntnap2*^*+/+*^ mice with seizures treated with AAV-S5E2-Meis2 or AAV-S5E2-dTom. **c.** Mean seizure duration for all groups show comparable length of seizures across groups.

**Fig. 5.**
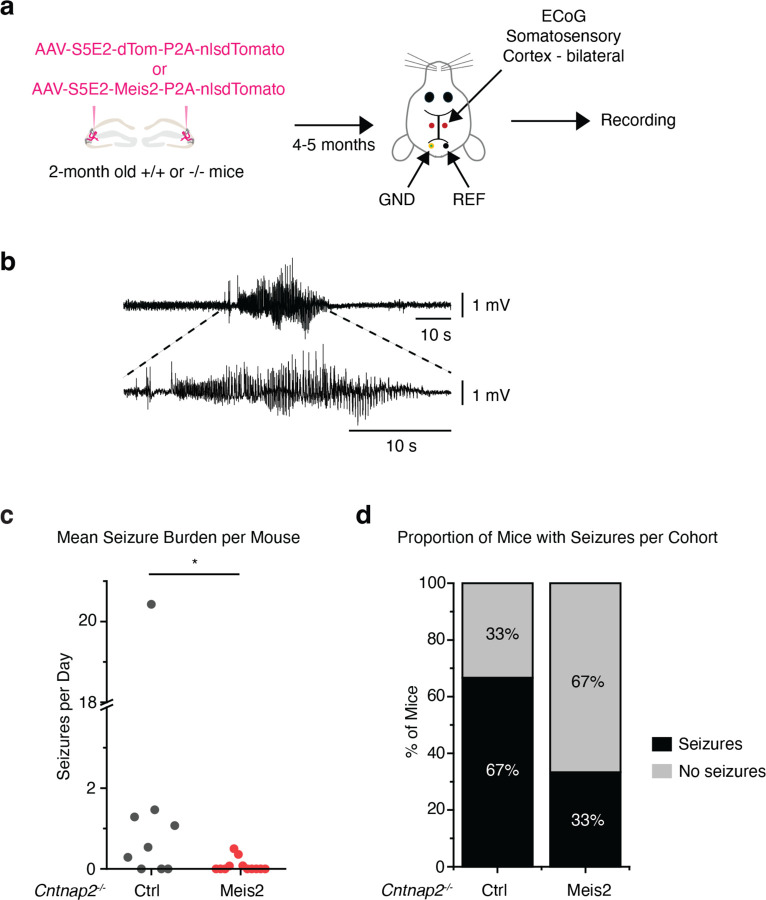
*Meis2*-dependent suppression of seizures in *Cntnap2* KO mice. **a**, Experimental timeline, AAV injections and ECoG implants (N = 9 for *Cntnap2*^−/−^ with Ctrl virus; 12 mice for *Cntnap2*^−/−^ with Meis2 virus). **b,** Representative seizure recorded in vivo from a *Cntnap2* KO mouse (top) and at an expanded timescale (bottom). **c,** *Cntnap2*^−/−^ mice injected with AAV-S5E2-Meis2 have significantly fewer seizures in the two-week recording period compared to mice injected with AAV-S5E2-dTom. * P < 0.05, Mann-Whitney Rank Sum. **d,** Proportion of *Cntnap2*^−/−^ mice with seizures treated with AAV-S5E2-Meis2 or AAV-S5E2-dTom. GND=ground, REF=reference.

*Cntnap2*^*−/−*^ mice develop seizures between 4–6 months of age^40,42,43^. 4–5 months following viral-mediated upregulation of *Meis2* in CA3/CA2 PV INs of 2 months old *Cntnap2*^*+/+*^ and *Cntnap2*^*−/−*^ mice, electrocorticography (ECoG) electrodes were implanted, and 24/7 chronic ECoG recordings were performed for 2 weeks after recovery from surgery (Fig. 5a). Seizure frequency and duration were quantified in all experimental groups. Two-thirds of *Cntnap2*^*−/−*^ mice (6 of 9 mice) treated with a control AAV (AAV-S5E2-dTom, 6 of 9 mice) displayed spontaneous electrographic seizures with a mean frequency of 2.79 (± 6.64 SD, ± 2.21 SEM) per day. These seizures displayed stereotypical preictal spiking, high amplitude synchronous activity, and a post-ictal depression, and they were associated with convulsive motor seizures. One-third of *Cntnap2*^*−/−*^ mice (4 of 12) treated with *Meis2* AAV (AAV-S5E2-Meis2, 4 of 12 mice) displayed spontaneous seizures with a mean frequency of 0.08 (± 0.16 SD, ± 0.04 SEM) per day. Boosting *Meis2* in CA3/CA2 PV INs significantly reduced seizure frequency in *Cntnap2*^*−/−*^ mice (Fig. 5b–d). We also examined *Cntnap2*^*+/+*^ mice and found no seizures in any *Cntnap2*^*+/+*^ mice treated with the control virus (AAV-S5E2-dTom, 0 of 3 mice) but we did identify one *Cntnap2*^*+/+*^ mouse treated with AAV-*Meis2* (AAV-S5E2-*Meis2*) that had 2 seizures during the 2-week recording window (1 of 6 mice)(Extended Data Fig. 8a–b). Seizure duration was similar across all groups (Extended Data Fig. 8c). These findings demonstrate that boosting *Meis2* in CA3/CA2 PV INs reduces the likelihood of seizures in *Cntnap2*^*−/−*^ mice.

## Discussion

Impaired experience-dependent refinement of inhibitory circuits contributes to development of NDDs including ASDs^18–20,25,26^. Loss of hippocampal PV IN functions is thought to increase risk of developing core features of ASDs. These include imbalanced excitation and inhibition, network hyperexcitability, seizures, and cognitive impairment through reduced perisomatic inhibition of principal cells, principal cell recruitment into ensembles, and generation of network oscillations. Exome sequencing has identified ultra-high confidence genes that underlie different NDDs including ASDs and schizophrenia (https://gene.sfari.org/)^34,35^ but how these different genes converge upon biological mechanisms that govern PV IN functions essential to cognition is poorly understood. Experience-dependent PV IN plasticity is a candidate biological mechanism for NDDs since it underlies the capacity of PV INs to coordinate changes in their intrinsic properties, synaptic physiology, input-output connectivity and synaptic plasticity in response to experiential demands. However, identities of cell-autonomous regulators of experience-dependent PV IN plasticity in adult hippocampus have remained elusive. We designed an input-specific activity sensitive screen for XPGs in CA3/CA2 PV INs of adult mice that does not rely on imposition of artificial patterns of activity onto PV INs but mimics physiological experience-dependent changes in mossy fiber inputs onto PV INs that trigger a change in PV IN cell state properties. We discovered a suite of XPGs including transcription factors and epigenetic regulators whose expression in PV INs is increased in response to increased mossy fiber excitatory drive. A significant proportion of upregulated, but not downregulated, XPGs in CA3/CA2 PV INs in our unbiased screen exhibit loss-of-function mutations in ASDs and epilepsies, and several encode ultra-high confidence risk genes for schizophrenia. Based on the directionality of expression change in XPGs in our screen and loss-of-function mutations in these genes in NDDs, we propose that impaired experience-dependent PV IN plasticity is a convergent mechanism for NDDs including ASDs and schizophrenia.

To test the hypothesis that loss of experience-dependent PV IN plasticity in CA3/CA2 is a substrate for cognitive impairments and seizures in NDDs, we chose the *Cntnap2* KO model of NDD risk. This was not to model a specific NDD, but because it exhibits a plethora of developmental brain-wide deficits including neuronal migration, myelination, neuronal excitability, loss of GABAergic inhibition and PV INs, cognitive impairments and seizures^40–43^. We characterized MEIS2 as a transcriptional regulator of experience-dependent PV IN plasticity in CA3/CA2 and demonstrated that *Meis2*-dependent restoration of experience-dependent PV IN plasticity in CA3/CA2 in adulthood in *Cntnap2* KO mice is sufficient to reverse developmental deficits in excitation-inhibition balance, cognitive impairments and seizure risk. MEIS2-dependent suppression of seizures may reflect coordinated reduction in excitatory and increased inhibitory synaptic transmission in DG-CA2 ^47^ and potentially, also in DG-CA3.

The effects of increasing levels of MEIS2 in CA3/CA2 PV INs on physiology, synaptic connectivity and plasticity reflect an amalgam of previously identified roles for MEIS2 in regulating GABAergic projection neuron identity in development and maintenance of sensory neuron physiology and terminal specialization^21,38,39^.We infer that MEIS2 may function as a permissive coactivator that acts in concert with other transcription factors to instruct different facets of experience-dependent PV IN plasticity. The observation that *Meis2* expression is not detected in the medial ganglionic eminence^8,21^, the embryonic source for hippocampal PV INs, suggests an evolutionarily parsimonious repurposing of *Meis2* based on function rather than PV IN identity specification, to regulate experience-dependent programs within PV INs in the adult brain. Whether boosting *Meis2* in cortical PV INs and cortical PV IN projection neurons has similar effects to those described in CA3/CA2 PV INs remains to be determined.

Our study provides a mechanistic framework to investigate how PV INs dynamically change their properties, physiology and connectivity, defining characteristics of cell state in response to experience to influence encoding of contextual, social and spatial information in DG-CA2/CA3. The spatial and temporal induction of XPGs in CA3/CA2 PV INs may enable DG to flexibly switch between two modes of CA3/CA2 principal cell activity regulation, namely global suppression of all principal cells through provision of PV IN mediated blanket inhibition^7^ or selective inhibition of distinct principal cells to dictate ensemble dynamics, remapping and generation of network oscillations. While evidence for the latter mode is scarce, a recent study demonstrated that cortical chandelier cells exhibit activity-dependent restructuring of axo-axonic synapses onto individual principal cells to improve direction tuning^48^. XPG-encoding transcription factors may contribute to dynamic regulation of PV IN mediated inhibition of CA2 (and CA3) neurons in the following ways. First, XPG-encoding transcription factors may recruit different modules of ion channels, cytoskeletal proteins, ubiquitin ligases, cell adhesion proteins, and axon guidance cues to modify PV IN spatial tuning, timing of inhibition, strength, number and distribution of perisomatic synapses to dictate recruitment of principal cells into neuronal ensembles and network oscillations. Upregulation of XPGs encoding ion channel subunits such as *Grin2a* may increase or restore PV IN excitability and action potential firing properties^49^,whereas the GluA2 subunit, *Gria2*, may dynamically calibrate PV IN spatial selectivity in response to increased mossy fiber inputs^17^. XPGs encoding axon guidance cues may sculpt the distribution of PV IN synapses across CA2/CA3 principal cells in an activity-dependent manner. For example, upregulation of secreted Semaphorin 3C in cortical PV INs reduces perisomatic PV IN synapses on principal cells^50^. Balanced regulation of XPGs is probably crucial for optimal PV IN functions since boosting *Meis2* in CA3/CA2 PV INs of *Cntnap2*^*+/+*^ mice impaired cognition. Second, different XPGs maybe upregulated by experience in distinct subsets of PV INs (vertical and horizontal basket cells, axo-axonic cells)^37^and through differences in output connectivity, regulating spiking of distinct subsets of principal cells. Third, signaling mechanisms and temporal kinetics of XPG induction may differ. Our analysis of *Tbr1* and *Meis2* provides support for these different facets of XPG regulation in PV INs. We show that these two transcription factors have overlapping but distinct effects on experience-dependent PV IN plasticity. Furthermore, *Tbr1* and *Meis2* are expressed at low levels in distinct hippocampal PV subtypes ^37^. Future studies mapping XPG induction in PV subtypes and deploying PV IN subtype specific enhancer driven viral vectors will help instantiate possibilities. Together, such efforts may illuminate how XPG-dependent combinatorial codes specifying experience-specific “PV IN cell states” expand the capacity for circuit and network computations in adult hippocampus and determine whether loss of distinct XPG-associated codes underlie specific circuit and network alterations that distinguish ASDs and schizophrenia.

In conclusion, we identify experience-dependent PV IN plasticity as a convergent biological mechanism for NDDs and demonstrate that restoring experience-dependent PV IN plasticity in adulthood offers an opportunity for therapeutic intervention in adulthood to alleviate cognitive impairments and reduces seizures in NDDs. Since the principal cell-PV IN-principal cell feed-forward inhibition motif is found in brain circuits supporting memory, cognitive flexibility, decision making and attention, targeting experience-dependent PV IN plasticity in these brain regions may reduce network excitability and impact different domains of cognition in NDDs.

## Methods

### Mice.

All mice were group housed and experiments were conducted in accordance with procedures approved by the Institutional Animal Care and Use Committees at the Massachusetts General Hospital and Tufts University School of Medicine and NIH guidelines. All mice were housed in a 12-h (7:00 a.m. to 7:00 p.m.) light–dark colony room at 22–24 °C with *ad libitum* access to food and water. *Cntnap2*^*−/−*^ mice, *Pvalb-cre* mice, and *Rpl22*^*HA*^ mice were obtained from the Jackson Laboratories (strain #017482, 017320, and 011029, respectively).

#### Viruses and virus constructs.

AAV-S5E2-dTom-nlsdTom plasmid and AAV PHP.eB virus were purchased from Addgene (plasmid #135630). AAV-S5E2-Meis2-nlsdTom virus was generated by subcloning mouse Meis2 into AAV-S5E2-dTom-nlsdTom plasmid (VectorBuilder). AAV-S5E2-dSACas9/VP64 was generated by subcloning mouse dSACas9/VP64 into AAV-S5E2-dTom-nlsdTom plasmid (VectorBuilder). AAV-U6-sagRNA#1#2-Syn1-P2A-mcherry and AAV-Herc1gRNA#1#2-Syn1-P2A-mcherry were generated by VectorBuilder. Cal-Light viruses: AAV-pCMV-Myc-TM-KA2-CaM-NES-TEV-N-AsLOV2-TEVseq-tTA, pAAV-hSYN-M13-TEV-C-P2A-tdTomato, pAAV-TRE-EGFP (gifts from Hyun Lab). AAV.PHP.eB viruses were produced by Boston Children’s Hospital Viral Core.

### Immunohistochemistry.

Mice were anaesthetized with ketamine and xylazine (10 mg/ml and 1.6 mg/ml, IP), transcardially perfused with 4% PFA, and brains were incubated in 4% PFA at 4°C overnight. Brains were placed in 30% sucrose/PBS for 2 days and then embedded in medium (OCT, Fisher HealthCare). 35 μm cryosections were obtained (Leica) and stored in PBS (0.01% sodium azide) at 4°C. For immunostaining, floating sections were permeabilized, blocked in blocking solution for 2 h (PBS containing 0.3 % Triton X-100 and 10% normal donkey serum, NDS), and then incubated with primary antibodies (PBS containing 10% NDS) at 4°C overnight. Sections were then washed with PBS 3 times, 10 min each, then incubated with secondary antibodies in PBS for 2 h at room temperature (RT). Sections were then washed with PBS 3 times, 10 min each, mounted on glass slides and coverslipped with mounting medium containing DAPI.

### Image analysis.

For PV puncta and synaptotagmin-2 puncta: Images were obtained from 3 sections per mouse hippocampus blind to treatment and genotype. A Leica SP8 confocal laser microscope and LAS software were used to capture images in the stratum lucidum at high-resolution (2,048). Single confocal plane images were captured in the CA2 and CA3ab subfields using a 63X oil objective plus 43X digital zoom. Quantification sample size: PV^+^ puncta or Syt2^+^ puncta, densities were averaged from 18 images per mouse. Puncta was analyzed using the StarDist 2D plugin and particle analysis tools in FIJI ImageJ. Threshold values were held constant across images. For Cal-Light, dCA3a cells were manually counted. To reduce contribution of background fluorescence, a threshold based on maximizing Yen entropy (https://ieeexplore.ieee.org/document/366472) was applied to GFP images during quantification.

### Antibodies.

PV (rabbit, Swant PV25, 1/5000; goat, Swant PVG213, 1/1000 ); RGS14 (mouse, NeuroMab 75–170, 1/500; rabbit, Proteintech 16258-1-AP, 1/500); Synaptotagmin 2 (mouse, Abcam AB154035–1001, 1/250); RFP (rabbit, Rockland 600-401-370, 1/1000; goat, Sicgen AB1140–100, 1/500); cFOS (guinea pig, Synaptic Systems 226–004, 1/3000); GFP (chicken, Invitrogen A10262, 1/500). Fluorescent-label-coupled secondary antibodies (Alexa-Fluor-488-conjugated donkey anti-rabbit IgG, 711-545-152, 1:500; Cy3-conjugated donkey anti-rabbit-IgG, 711-165-152, 1:500; Alexa-Fluor-488-conjugated donkey anti-mouse-IgG, 715-545-151, 1:500; Cy3-conjugated donkey anti-mouse-IgG, 715-165-151, 1:500; Alexa-Fluor-488-conjugated donkey anti-chicken-IgG, 703-545-155, 1:500; Cy3-conjugated donkey anti-goat-IgG, 705-165-147, 1:500; 647-conjugated donkey anti-guinea pig-IgG, 706-605-1481; Cy3-conjugated donkey anti-guinea-pig-IgG, 706-165-148, Jackson ImmunoResearch, in 1/500 dilution.

### RNA in situ hybridization and immunofluorescence imaging.

RNAscope in situ hybridization was performed following the RNAScope Multiplex Fluorescent Reagent Kit v2 protocol (323100) (Advanced Cell Diagnostics, ACD). Briefly, mice were perfused with PBS and followed by 4% PFA. Brain slices (10 um) were mounted and dried at −20 °C for 2 hours. The slides were washed in PBS, then dehydrated in a gradient of ethanol (50%, 70%, 100%, and 100%) for 5 min each and air-dried for 5 min. RNAscope Hydrogen Peroxide was applied to tissues and incubated at RT for 10 min, followed by treatment with RNAScope 1X Target Retrieval Reagent. Tissues were incubated for 10 min at 95 °C followed by washed in ddH2O and 100% ethanol. Tissues were dried at RT for 3 min. RNAScope Protease III was applied and incubated for 30 min at 40 °C, then washed with water. The target probes were prepared (MmRgs14, 416651; Mm-Pvalb-C2, 421931-C3; Mm-Meis2-C3, 436371-C3). Target probes were applied and incubated at 40 °C for 2 h, after which tissues were washed with 1X wash buffer and placed in 5X SSC buffer overnight. Tissues were washed with wash buffer twice followed by incubation with AMP1, AMP2, and AMP3 (30 min for AMP1 and AMP2, 15 min for AMP3, at 40 °C). Tissues were washed with wash buffer twice in between each amplification step, then incubated with HRP specific to each channel (15 min 40 °C). Tissues were washed twice, after which the TSA fluorophore specific to each channel/probe (Opal 520, 570, and 690, Akoya Biosciences)was added and incubated for 30 min at 40 °C). Tissues were washed twice and incubated with RNAScope HRP Blocker for 15 min at 40 °C. Fluorescent images were captured using an SP8 Leica confocal microscope. All Pvalb^+^ cells were outlined and *Meis2* intensity were quantified.

### RNA sequencing

#### Tissue collection and RNA isolation

Hippocampal CA2/CA3 regions were harvested following injections of *Ablim3* shRNA or non-target RNA-expressed lentiviruses into the DG of 2-month old *PV-Cre*:*Rpl22HA*^*f/f*^ mice for 2 weeks. Brain tissues were snap-frozen and pooled from 6 mice (male and female) for every sample. Samples were immunoprecipitated with anti-HA magnetic beads (Pierce: 88836) for 3 hours at 4 °C ^51 32^. After elution with RNeasy Plus Micro Kit (Quiagen), purified ribosome-associated RNAs were stored at −80 °C. RNA quality was assessed on a TapeStation (Agilent) and RNA amounts were quantified using the Qubit 4.0 Fluorometer (Life Tech.). Only RNA samples with RIN above 8.0 were used for library preparation and sequencing.

#### RNA-seq

RNA-seq libraries were constructed from total RNA using Clontech SMARTer v4 kit (Takara), followed by sequencing on an Illumina HiSeq 2500 instrument, resulting in 20–30 million 50 bp reads per sample. The STAR aligner ^52^ was used to map sequencing reads to transcriptome in the mouse mm9 (GRCm37) reference genome. Read counts for individual genes were produced using the unstranded count function in HTSeq v.0.6.0 ^53^, followed by the estimation of expression values and detection of differentially expressed transcripts using EdgeR ^54^ and included only the genes with count per million reads (CPM) 1 for one or more samples ^55^. Differentially expressed genes were defined by at least 1.5-fold change with FDR< 0.05.

### qRT-PCR analysis.

Hippocampal DG or CA2/CA3 regions were harvested and snap-frozen (https://www.jove.com/video/1543/dissection-of-hippocampal-dentate-gyrus-from-adult-mouse). Briefly, cDNA samples reverse transcribed from RNA collected from *PVCre:Rpl22*^*HA/HA*^ mice with either lenti-shNT or shRNA (*Ablim3*) injection into DG. Total RNA was quantified using a NanoDrop spectrophotometer (Thermo Scientific) and then equal amounts of RNA were used for reverse transcription (SuperScript IV First-strand synthesis system, Invitrogen). qRT-PCR was carried out with SYBR green (BioRad) and primers (Primer bank)^56^ with following sequences: *Ablim3*-F 5’-GGTCCGTGTCCACAACAAC; *Ablim3*-R 5’- GTCCCGGCAGCTATCACAG; *Bok*-F 5’-CCACAGACAAGGAGCTGGT; *Bok*-R 5’- TAGCCAAGGTCTTGCGTACA; *Dsp*-F 5’-CGGACATTCATGCGAGATAC; *Dsp*-R 5’- GCCTTGAACTGGGAACACTC; *Bcl11a*-F 5’-TGGTATCCCTTCAGGACTAGGT; *Bcl11a*-R 5’- TCCAAGTGATGTCTCGGTGGT; *Meis2*-F 5’-CAGGGTGGTCCAATGGGAATG; *Meis2*-R 5’- GGGGGTCCATGTCTTAACTGAG; *Tbr1*-F 5’-CAAGGGAGCATCAAACAACA; *Tbr1*-R 5’- GTCCTCTGTGCCATCCTCAT; *Herc1*-F 5’-GAAGATGTGGATGCAGCAGA; *Herc1*-R 5’- GGTCTGTCCGGTGAAGGATA; *Gapdh*-F 5’-GCTT GTCATCAACGGGAAG; *Gapdh*-R 5’-TTGTCATATTTCTCGTGGTTCA.

### GSEA.

Gene Set Enrichment Analysis was performed using the GSEA package (https://www.gsea-msigdb.org/gsea/index.jsp) against mouse MSigDB v2024.1.Mm collection of pathways ^57,58^, with default settings and the cutoff of false discovery rate (FDR) < 0.05.

### Stereotactic viral injection.

Mice were administered carprofen (5 mg/kg, SQ) before surgery and were then anaesthetized with ketamine and xylazine (10 mg/ml and 1.6 mg/ml, IP). Mice were placed in a stereotaxic frame, and a small hole was drilled at each injection site (Foredom K.1070 High Speed Rotary Micromotor Kit). Bilateral injections were performed using Hamilton microsyringes (Hamilton, Neuros Syringe 7001) or Nanoject digital microinjectors that were slowly lowered into target sites and remained in place for 8 min prior to viral infusion at a rate of 50 nl/min. The coordinates relative to bregma: dorsal DG: –1.8 mm (AP), ±1.35 mm (ML), –2.25 mm (DV) and dorsal CA2/CA3: –1.8 mm (AP), ±2.45 mm (ML), –2.35 mm (DV). Recombinant AAVs (titre: 1×10^13^) were injected for a total volume of 100 nl per injection site. 10 minutes after infusion, the microsyringes were slowly withdrawn and the skin above the incision was sutured with coated vicryl sutures. For Cal-Light ^46^, viruses were mixed (1:1:1 ratio) and injected for a total volume of 300 nl. One week later, AAV-control or AAV-Meis2 virus were injected for a total volume of 100 nl per injection site. The coordinates relative to bregma: AP −1.80, ML ±2.28, DV −2.20 for virus injection; AP −1.80, ML ±2.28, DV −1.80 for optical fiber probe implant). Post-surgery, mice were placed in a clean empty cage on top of a heating pad with ambient temperature to 36°C until full recovery from anesthesia. Mice were monitored and received a daily injection of carprofen (5 mg/kg, IP) for 3 days following surgery ^9^.

### Behavioral procedures.

Two weeks after viral injections, mice were handled for 3 days prior to behavioral experiments to habituate them to human handling and transportation from vivarium to behavioral testing rooms. The behavioral assays were performed in the following order: open-field (OF, day 1), visual cue habituation (day 2), novel object location followed by novel object recognition (NOR, day 3), and social recognition and discrimination (day 4). All the behavioral assays were performed in the same chambers (40 × 40 cm, MazeEngineers). Videos were recorded and exported from Freezeframe (Actimetrics) and analyzed with EthoVision XT 15 (Noldus). Center point tracking was used to record movement and nose point tracking was used to evaluate object and social interaction. An interaction was registered when the test mouse’s nose position to object or stimulus mouse was within 1 cm ^9^.

#### Behavioral paradigm for PV IN synapse/Syt2/puncta analysis:

Mice were handled for 7 days prior to behavioral experiments to habituate them to human handling, transportation from vivarium to behavioral testing rooms and the context for one hour. On Day 8, mice were either exposed to context only or with one stimulus mouse in one pencil cup for 10 min. Mice were returned to their home cage and subsequently perfused 90 minutes later.

#### Behavioral paradigm for *Meis2* intensity analysis:

Mice were handled for 3 days prior to behavioral experiments to habituate them to human handling, transportation from vivarium to behavioral testing rooms and the context for one hour. On Day 4, mice were either exposed to context only or with one stimulus mouse in one pencil cup for 10 min. Mice were returned to their home cage and subsequently perfused 10 minutes later.

### Open-field paradigm.

Mice were transported into a holding room and habituated for one hour prior to testing. Total distance traveled and the time spent in the center of the arena were quantified over 30 min^9^.

### Novel object location and recognition.

Two identical objects (2 × 4 × 6 cm) were placed in the OF chamber along one side (5 cm distance from the wall). Mice were placed in the opposite side of the object and allowed to explore freely for 5 min and then returned to their home cage for 2 hrs. Then one object was moved to the opposite side, and mice were placed in the middle of objects and allowed to explore for 5 min. Mice were returned to home cage for 10 min. Next, one object was replaced with a novel object (4 × 4 × 6 cm, 5 cm distance from the wall), and mice were placed in the middle of the objects and allowed to explore freely for 5 min. The time spent exploring the objects (noise point within 2 cm) was quantified^9^.

#### Social recognition and social discrimination.

Stimulus mice (strain, age, and sex-matched) were habituated to being placed in a pencil wire cup in the OF chamber prior to the task day for 3 days, 15 min per day. The task consisted of 3 trials: habituation (empty cup vs empty cup), recognition (empty cup vs stimulus), and discrimination (novel vs familiar), with 5 min intertrial intervals. Subjects were placed in the center of the chamber for 10 min. The locations of the familiar and novel stimuli were counterbalanced across trials. The time spent exploring the stimulus mouse was quantified (nose point within 1 cm) ^9^.

### Tagging socially active neurons by soma-targeted Cal-light.

We used the soma-targeted Cal-Light system^46^ to tag active cells in the CA3a region of the hippocampus during social interaction. Mice were bilaterally injected in CA2/3 with a CAL-light viral mix three weeks prior to behavioral testing. One week after injection, animals were implanted with an optical fiber probe above CA2/3 and allowed a two-week recovery before behavioral testing.

#### Behavioral paradigm and tagging protocol:

Before testing, animals were placed in holding cages for a minimum of one hour, using the same cage each day. Subject mice underwent a three-day habituation protocol, during which they explored an open-field chamber with two empty cups placed in opposite corners while attached to lightless fiberoptic cables. One day following habituation, subject mice were exposed to a novel stimulus mouse (stimulus 1) placed under one of the cups and allowed to explore the chamber for 10 minutes. When subjects approached within 5 cm of the stimulus mouse, blue light was delivered through fiber optic cables to mediate Cal-Light tagging of the engram. Immediately after the session, subjects were returned to their holding cages. Four hours after initial exposure, subjects were reintroduced to the chamber for a second 10-minute session, with a familiar mouse under the same cup as before. Following this, subjects were returned to holding cages and subsequently perfused 90 minutes later.

#### Optical fibers and laser delivery:

Optical fibers were constructed using a 200 μm core, 0.37 numerical aperture multimode fiber from Thorlabs. Fibers were inserted through a 230 μm core zirconia ferrule 701 (Precision Fiber Products) and glued in place before thoroughly polishing for a smooth connection between fiber and optical cable from the laser. For CAL-Light activation, a 100 mW 475 nm blue laser diode was used (OEM Laser Systems). When the subject animal was within a predefined zone, the light was delivered at a frequency of 1 KHz and an intensity of 5–7 mW using an external arbitrary waveform generator (Agilent) attached to the signal port of the laser.

### Ex vivo electrophysiology.

Mice were unilaterally injected with 0.3 µL pAAV5-CamKIIa-hChR2-eYFP into the dorsal DG, and bilaterally injected with 0.3 µL AAV-S5E2-Meis2-P2A-nlsTdtomato or AAV-P2A-nlsTdtomato control virus along the stratum lucidum mossy fiber pathway adjacent to CA2/CA3a pyramidal cell layer of the dorsal hippocampus. Mice were 2–3 months old prior to viral transfection (see above for stereotactic coordinates). At 2–3 weeks after viral infusion, mice were anaesthetized with ketamine and xylazine (10 mg/ml and 1.6 mg/ml, IP) then transcardially perfused with ice-cold (4 ºC) choline chloride-based artificial cerebrospinal fluid (ACSF) composed of (in mM): 92 choline chloride, 2.5 KCl, 1.25 NaH2PO4, 30 NaHCO3, 20 HEPES, 25 glucose, and 10 MgSO4·7H2O. Their brains were rapidly extracted following decapitation. Coronal slices (300 µm thick) containing the dorsal hippocampus were cut in ice-cold (4 ºC) choline chloride ACSF using a Leica VT1000 vibratome (Leica Biosystems) and transferred to warm (33 ºC) normal ACSF for 30 min. Normal ACSF contained (in mM): 124 NaCl, 2.5 KCl, 1.25 NaH2PO4, 24 NaHCO3, 5 HEPES, 12.5 glucose, 2 MgSO4·7H2O, 2 CaCl2·2H2O. All ACSF solutions were adjusted to a pH of 7.4, mOsm of 305, and were continuously saturated with carbogen (95% O2 and 5% CO2). Slices were allowed to cool to room temperature (20–22 ºC) for 1 hour before recordings.

Whole-cell patch-clamp recordings were amplified, low-pass filtered at 1.8 kHz with a four-pole Bessel filter, and digitized (Muliclamp 700B, Digidata 1550B, Molecular Devices). Slices were placed in a polytetrafluoroethylene submersion chamber and continually perfused with normal ACSF (>2 mL/min). Neurons were visually identified by infrared differential interference contrast imaging combined with epifluorescence using LED illumination (pE-300 white, CoolLED). Pyramidal neurons in CA2 and CA3ab were distinguished by their anatomical location and distinct electrophysiological properties. Borosilicate patch pipettes had a resistance of 4–5 MΩ and filled with an internal solution containing (in mM): 120 CsMeS, 4 MgCl2, 1 EGTA, 10 HEPES, 5 QX-314, 0.4 Na3GTP, 4 MgATP, 10 phosphocreatine, 2.6 biocytin, pH 7.3, 290 mOsm. For current-clamp recordings, patch pipettes were filled with 130 mM potassium gluconate in place of CsMeS and QX-314 excluded. Once GΩ seal was obtained, neurons were held in voltage-clamp configuration at −70 mV and the input resistance, resting membrane potential, and capacitance were measured. Series resistance (<30 MΩ) was monitored throughout recordings and recordings were discarded if series resistance changed by >20% from baseline.

Excitatory and inhibitory postsynaptic current (EPSC and IPSC) were optically evoked with 1 ms 473 light pulses delivered above the mossy fiber pathway – the hilus of the DG. Current responses were recorded at 1.5 × threshold, defined as the minimum stimulation intensity required to produce a consistent current response beyond baseline noise. Isolation of EPSC was done by voltage clamp at - 70 mV and IPSC at 0 mV. Optical 10 pulse stimulation trains were evoked 5 times at an interval of 20 sec between trains. The interevent interval between pulse stimulation was 100 ms.

Long-term potentiation (LTP) of the EPSC and long-term depression of the IPSC (iLTD) was induced by electrically evoked theta-burst stimulation (TBS) which consisted of bursts of 4 pulses at 100 Hz, interburst interval was 200 ms, and repeated 4 times at 10 sec intervals. A bipolar tungsten electrode in a patch pipette with a resistance of 1–2 MΩ and filled with ACSF was placed on the mossy fiber pathway – the hilus of the DG. Stimulation was set to 1.5 × threshold of the EPSC. The recording consisted of a 10 min baseline of the optically evoked EPSC or IPSC, TBS, then 40 min of the optically evoked postsynaptic current. Plasticity was calculated by the percent change from the last 5 min of the baseline current amplitude to the last 5 min of the 40 min recording.

Current clamp configuration was used to record intrinsic membrane properties of PV INs. To assess action potential spiking activity, the neuron was clamped at −70 mV and 10 pA ascending step currents were delivered at 500 ms durations. Bursting neurons were identified by their asynchronous rapid firing within the first 50 ms of the current step followed by failure to fire and were excluded from analyses. Spontaneous excitatory and inhibitory postsynaptic current (sEPSC and sIPSC) were recorded by voltage clamp at −70 mV for sEPSCs and 0 mV for sIPSCs. Pharmacological validation were conducted to ensure no sIPSCs were visible during sEPSC recordings and vice versa (data not shown). Briefly, no inward or outward events were detected after bath application of cyanquixaline (CNQX, 20 µM), an AMPA/kainite receptor competitive antagonist while voltage clamped at −70 mV. Likewise, no events were detected after bath application of gabazine (10 µM), GABAA receptor antagonist while voltage clamped at 0 mV.

Miniature EPSC and IPSC (mEPSC and mIPSC) were recorded after bath application of tetrodotoxin (TTX, 1 µM). Isolation of mEPSC was done by voltage clamp at −70 mV and mIPSC at 0 mV. Autodetection parameters for inclusion of spontaneous and miniature events was determined by calculating minimum threshold: root mean square (RMS)2 × 1.5. Data acquisition was performed using Clampex and analyzed with Clampfit (Molecular Devices) and EasyElectrophysiology (V2.5.2) software.

### Electrocorticography (ECoG) surgical implant.

All animal surgeries and subsequent experiments were conducted in accordance with Tufts University’s Institutional Animal Care and Use Committee guidelines and animal use protocols. 6–7-month-old *Cntnap2*^−/− and +/+^ mice that had previously undergone CA2/CA3 AAV injection were anesthetized with isoflurane (3% induction, 1.5% for maintenance, 2 l/min O2 flow rate) and given systemic analgesic (buprenorphine, 0.1 mg/kg, subcutaneous [SC]) as well as local analgesia near the incision site (bupivacaine, 4 mg/kg, SC). Mice were then placed in a stereotactic frame and the dorsal side of the skull was shaved and disinfected with iodine and ethanol. A scalp incision was made, and the skull surface cleaned and dried. Four burr holes were drilled into the skull without puncturing the dura mater. Drill bit tip diameter = 0.7 mm (Item No. 19007–07, Fine Science Tools, CA). Stereotaxic coordinates of the four drill holes: Anterior burr holes were −0.6 mm (bregma, AP axis) and 2 mm left (ECoG) and right (ground) of the midline (ML), posterior burr holes were – 2.6 mm (bregma, AP) and 2.5 mm left (reference) and right (ECoG) to ML. Four 0.1’’ stainless steel screw electrodes with attached silver wires (Part # 8403, Pinnacle Technologies, Wyckoff, NJ, USA) were gently screwed into the burr holes and fixed with dental cement and superglue to increase implant stability for chronic ECoG recordings. The silver wires of the electrode screws were then soldered to the headmount (Part # 8402, Pinnacle Technologies, Wyckoff, NJ, USA). The headmount/electrode construct was then secured to the skull with dental cement. After surgery, animals were allowed to recover for at least seven days before chronic ECoG recordings started and were given analgesia (Buprenorphine, 0.1 mg/kg, SC) as needed for the first 3 days after surgery.

### Chronic ECoG recording.

After recovering from surgery, a preamplifier (100 x gain, 1 Hz high-pass filter, Part # 8213, Pinnacle Technologies, Wyckoff, NJ, USA) was plugged into the implanted headmount and preamplifiers were attached to a commutator in the ECoG recording system (Pinnacle Technologies). Mice were housed in a round, acrylic ECoG recording chamber with access to food and water and kept on a standard light/dark cycle. ECoG data was recorded using LabChart Pro software (AD instruments, Colorado Springs, CO, USA) with a 1 kHz sampling rate. ECoG data was collected continuously 24 hours a day for at least 14 days. After recording, ECoG signals were filtered (100 Hz low-pass filter) and recordings were manually reviewed manual by an experienced, blinded investigator. When seizures were identified, their duration was quantified, and their time of incidence was noted.

### SLEAP.

Mice were pair-housed for over two weeks prior to recording. On the recording day, the cage lid was removed, and the cage was placed inside a recording chamber overnight with access to food and water. Behavior was continuously recorded for 8 hours under infrared lighting, with a one hour segment, captured one hour after the cage was placed in the chamber, selected for analysis. We used SLEAP (https://www.nature.com/articles/s41592-022-01426-1) to track and estimate the poses of freely behaving mice in their home cages. To analyze social interactions between mice, a multi-instance, bottom-up model with a U-Net architecture was trained on 2,751 instances across 917 frames, with 306 validation instances from 102 frames. Six body parts were labeled as nodes: nose, right ear, left ear, head, body center, and tail base. Our average distance between the predicted and ground truth nodes was 4.3 pixels. Training frames were randomly selected from videos containing different mice, with slight variations in contrast between subjects and background. Recordings were captured continuously during a 1-hour session, and the frame rate was 23.97 frames per second (fps). A batch size of 6 was used for training, with data augmentation through rotation (360°).

For inference, identity tracking across frames was performed using a flow tracker, with centroid similarity and the Hungarian matching algorithm applied over an elapsed frame window of 4 frames. Instances were manually inspected for identity switches and corrected before data export. Data were analyzed using a Python script modified from SLEAP analysis examples (https://sleap.ai/notebooks/Analysis_examples.html ) was used to analyze social interactions based on the distance between nodes. First, Missing nodes were linearly interpolated, and predictions were smoothed using a Savitzky-Golay filter to reduce jitter. We then measured nose-to-nose, nose-to-center, and nose-to-rear distances. Interactions were defined by pixel thresholds: 61 pixels (nose-to-nose), 100 pixels (nose-to-center), and 70 pixels (nose-to-rear). Thresholds were verified against manually scored data to ensure accuracy.

### Sex as a biological variable.

Sex of all mice was factored into design and analyses. If no statistical difference or interaction between sex were observed, then mice were grouped and analyzed according to experimental condition.

### Statistics, rigor, and reproducibility.

All experimenters were blind to treatment conditions throughout data collection, scoring, and analyses. Statistical analyses were conducted using Prism v10 (GraphPad) and the minimum sample size was determined based on prior experience of experimental paradigms, existing literature, and power analyses. Statistical significance was defined as *p* < 0.05. Appropriate nonparametric tests were used when data sets failed to meet parametric assumptions. Grubbs’ or ROUT tests were used to identify outliers with α or Q = 0.05. Detailed statistical analyses can be found in Supplementary Table 2.

## Extended Data

## Supplementary Material

Supplement 1 Supplementary Table 1
Complete gene list including DEGs
Supplementary Table 2
Complete Statistics table
