# Supplementary material for: Pro-cognitive restoration of experience-dependent parvalbumin inhibitory neuron plasticity in neurodevelopmental disorders
Source: Res Sq. 2025 Jan 16:rs.3.rs-5624085. Preprint. [Version 1] doi: 10.21203/rs.3.rs-5624085/v1 (PMC11774442; doi:10.21203/rs.3.rs-5624085/v1)
Supplement: Supplement 1 — Supplementary Table 1 Complete gene list including DEGs Supplementary Table 2 Complete Statistics table [file NIHPPRS5624085v1-supplement-1.pdf]

Supplementary Files

This is a list of supplementary files associated with this preprint. Click to download.

- SupplementaryTable1.AllGenescpmshRvsNT.xlsx
- SupplementaryTable2CompleteStatistics.xlsx
